# Supplementary material for: Dissecting the effect of long-term exposure to air pollution on risk of dementia in UK Biobank
Source: Environ Health. 2025 Dec 29;24:96. doi: 10.1186/s12940-025-01243-x (PMC12751783; doi:10.1186/s12940-025-01243-x)
Supplement: Supplementary file 1 — Supplementary Material 1. [file 12940_2025_1243_MOESM1_ESM.pdf]

Dissecting the effect of long-term exposure to air pollution on risk of dementia in UK Biobank Supplementary Information

Contents

1 UK Biobank variables **2**
1.1 Outcomes . . . 2
1.2 Exposure . . . 2
1.3 Covariates . . . 2

2 Missing data **4**

3 Alzheimer's disease and vascular dementia outcomes **5**

4 Mixture exposure analysis **11**

5 Variable selection analysis **12**

6 Recruitment centre random effects **13**

7 Positive control analysis **15**

8 Negative control analysis **19**

9 Control for noise pollution **20**

10 Effect modification by deprivation level **21**

11 Proportionality assumption check **25**

12 Air pollutants characteristics **29**

13 Variation inflation factor **32**

1 UK Biobank variables

Number within parentheses indicates the UK Biobank data field for the associated variables.

1.1 Outcomes

We used algorithmically defined outcomes for all-cause dementia (UK Biobank field 42018), Alzheimer's disease (AD, 42020), vascular dementia (VAD, 42022), and chronic obstructive pulmonary disorder (COPD; 42016) data. ICD 9/10 codes are (also listed in `https://biobank.ndph.ox.ac.uk/showcase/refer.cgi?id=460`): 290.4, F01, F01.0, F01.1, F01.2, F01.3, F01.8, F01.9, and 167.3 for vascular dementia, 331.0, F00, F00.1, F00.2, F00.3, F00.9, G30, G30.0, G30.1, G30.8, G30.9 for Alzheimer's disease, and all these plus 290.2, 290.3, 291.2, 294.1, 331.1, 331.2, 331.5, A81.0, F02, F02.0, F02.1, F02.2, F02.3, F02.4, F02.8, F03, F05.1, F10.6, G31.0, G31.1 and G31.8 for all-cause dementia. Oily fish intake frequency data were collected at baseline with the question 'How often do you eat oily fish?' (1329), to which participants could respond with 'Never', 'Less than once a week', 'Once a week', '2-4 times a week', '5-6 times a week', 'Once or more daily', ' Do not know' and 'Prefer not to answer'; we encoded '2-4 times a week', '5-6 times a week' and 'Once or more daily' as '$>$ 2 per week', because the latter two responses accounted for only $\sim 1\%$ or the responses, whereas 'Do not know' and 'Prefer not to answer' were considered as missing. In order to preserve the direction of the association detected between air pollution, index of multiple deprivation (IMD) and dementia incidence, when instead using fish intake frequency, the order of the encoding was as follows: '$>$ 2 per week' $<$ 'once a week' $<$ '$<$ once a week' $<$ 'never'.

1.2 Exposure

We used estimates of individual residential address exposure to $PM_{2.5}$ (24006), $PM_{absorbance}$ (24007), $PM_{2.5-10}$ (24008), PM_{10} (24005), NO_2 (24003) and NO_x (24004) for the year 2010. Exposure estimates were obtained by linking each participant's residential address to the annual average concentrations of air pollution derived at a $100m^2$ resolution from land use regression (LUR) models, as part of the European Study of Cohorts for Air Pollution Effects (ESCAPE) [1, 2]. In that study, NO_x was measured using a passive samplers with a filter for both NO and NO_2; thus, NO measures could be obtained by subtracting NO_2 from NO_x levels [3]. In the UK, the output of the LUR models were tested using data from air pollution monitors placed in the London/Oxford and Manchester areas, hence estimates are valid up to 400 km from Greater London. Consequently, all participant with addresses outside this area have missing data, and were therefore excluded from the analysis (Figure 1 in the manuscript). Air pollution data from the ESCAPE project have been shown to match well with data derived from UK air information resources [4, 5].

1.3 Covariates

Information about age (21022), sex (31), ethnicity (21000), educational attainment (6138), household income (738) and recruitment centre (54) was collected through baseline questionnaires or based on location of assessment centre. Age was encoded as a continuous variable, sex and recruitment centre as categorical. For ethnicity, we categorised participants as 'white' if they replied 'White', 'British', 'Irish', 'Any other white background', or as 'other' for everything else. For educational attainment, we created an educational score based on years of education following Okbay[6], except that we capped the score for national vocational qualification (NVQ), higher national diploma (HND) and higher national certificate (HNC) at 19, so that is was less than 20 for college. Additionally, participants who replied 'do not know', 'prefer not to say', or did not reply were assigned the average score, whereas participants who replied 'none of the above' were assigned the minimum score of 7. For household income, we categorised participants as '$< 18,000$', '18,000-30,999', '31,000-51,999', '52,000-100,000', and '$> 100,000$'.

Other covariates were computed based on the participants home postcode, including population density (20118), IMD for England (26410) and average 24-hour sound level in dB of noise pollution (24024). Information about population density was derived from data generated from the 2001 census from the Office of National Statistics, and participants were categorised as 'urban' or 'rural', reflecting the meaning assigned to each code in the UK Biobank showcase (`https://biobank.ndph.ox.ac.uk/showcase/coding.cgi?id=91`). The IMD is a compound measure of deprivation at a small area level reflecting deprivation in seven distinct domains, including income, employment, health/disability, education/training, barriers to housing/services, environment and crime. Here we encoded deprivation categorically as quartiles, with the 1st quartile as reference. Noise estimates for year 2009 were modelled using a Common Noise Assessment Methods in Europe (CNOSSOS-EU) noise model [7]; noise pollution was encoded as a continuous variable.

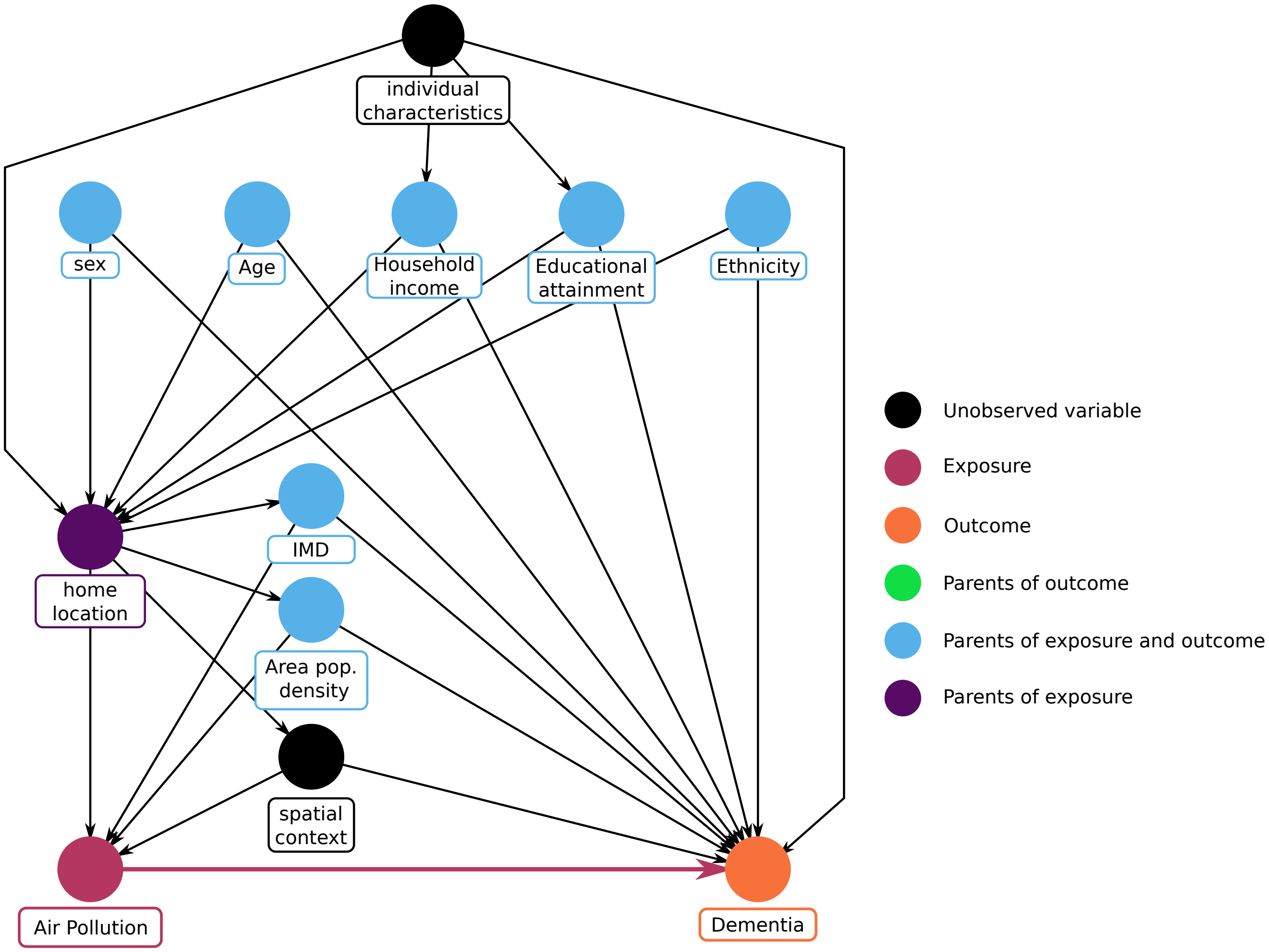

Figure 1: Direct acyclic graph. Index of multiple deprivation (IMD)

2 Missing data

In the UK Biobank study, missing data for air pollution (n=41286) exposure are only due to the participants living outside the area within which LUR model estimates are considered valid (up to 400 km from Great London); this mechanisms is likely to cause missing completely at random data in the context of assessing the effect of air pollution exposure on dementia incidence. On the other hand, the mechanisms underlying missingness for the other baseline covariates, and in particular for income, which accounted for most missing data (86%), possibly depend on the covariates themselves (missing not at random mechanism). Because of this, we chose to perform a complete case analysis, which in this scenario would give unbiased results, assuming no other unobserved variable is associated with missing mechanisms [8]. After exclusion due to missing pollution exposure and pre-existing dementia, missing data for ethnicity, educational attainment, income, and population density were respectively 2400, 4570, 71696 and 4230; for English IMD, after excluding participants with IMD for Scotland and Wales, the number of missing values was 12447, most of which were due to missing Government's data (`https://biobank.ndph.ox.ac.uk/showcase/refer.cgi?id=6810`). Among participants with pollution and IMD data, 95% had an English IMD, and $\sim 5\%$ had a Scottish or Welsh IMD. The number of participants dropped because of missing pollution or covariate values was 146013, leaving 356115 out of 502128 participants.

3 Alzheimer's disease and vascular dementia outcomes

We used Cox regression to assess whether air pollution affects time to dementia occurrence. Figure 2 and 3 show the results for the pollution score and single-pollutant models with AD and VAD as an outcome, respectively. These models were adjusted for age, sex, ethnicity, educational attainment, income, and population density. Air pollution exposures were encoded either continuously, scaled by inter-quartile range (iqr), or categorically, as quartiles (2q, 3q, 4q); for categorical variables, the reference was the 1st quartile (not shown). For the (restricted) pollution score models, the exposure was the first principal component capturing variability in exposure to $PM_{2.5}$, PM_{abs}, NO_2, and NO.

Figure 4 and 5 show the results for AD and VAD, respectively, when using models additionally adjusted for IMD. Figure 6 and 7 show the results for AD and VAD, respectively, when additionally excluding participants who lived less than 5 years (instead of 1 year) at baseline address.

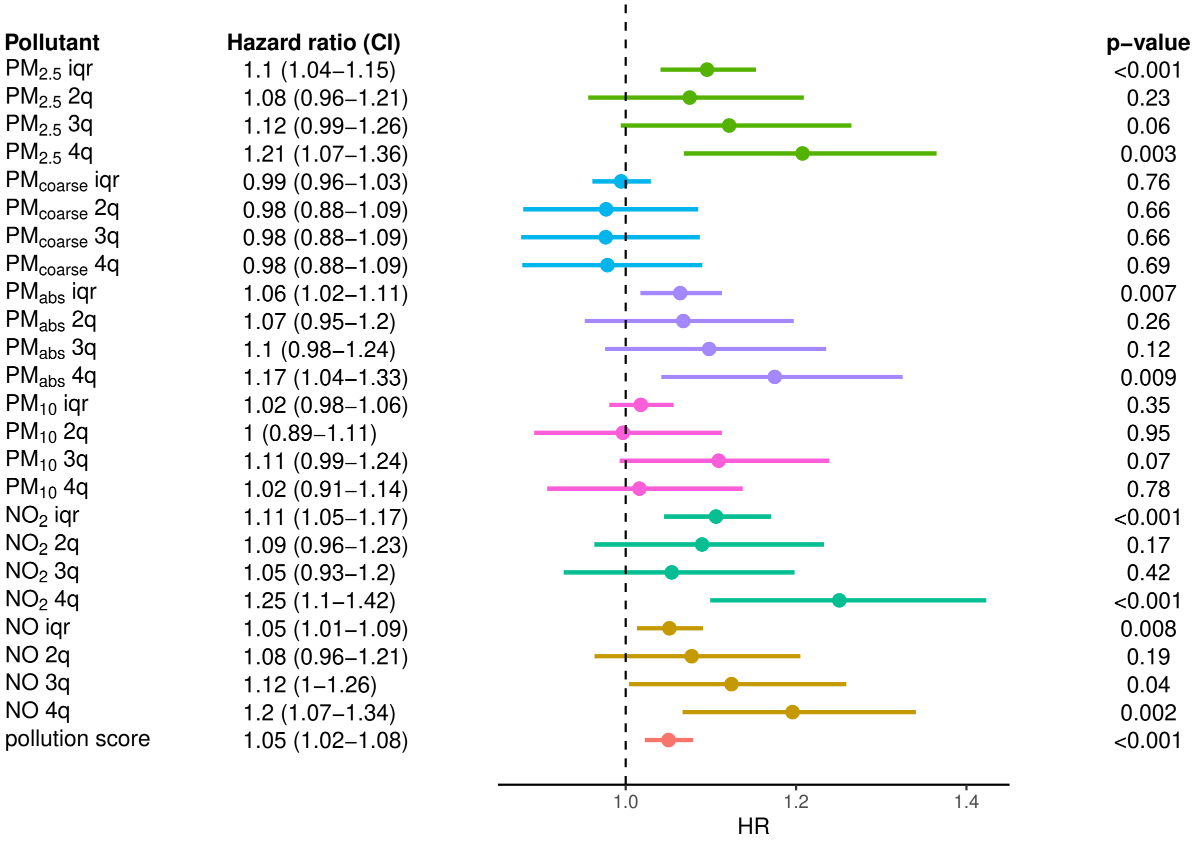

Figure 2: Effect of air pollution on AD. From left to right, for each air pollution exposure we show its associated hazard ratio and confidence interval, the forest plot and p-value of the effect estimate (Wald test); 'iqr' and 'n^{th}q' refer to the continuous (scale by IQR) and discrete exposures (n^{th} quartile), respectively. The models were adjusted for age, sex, ethnicity, educational attainment, income, and population density.

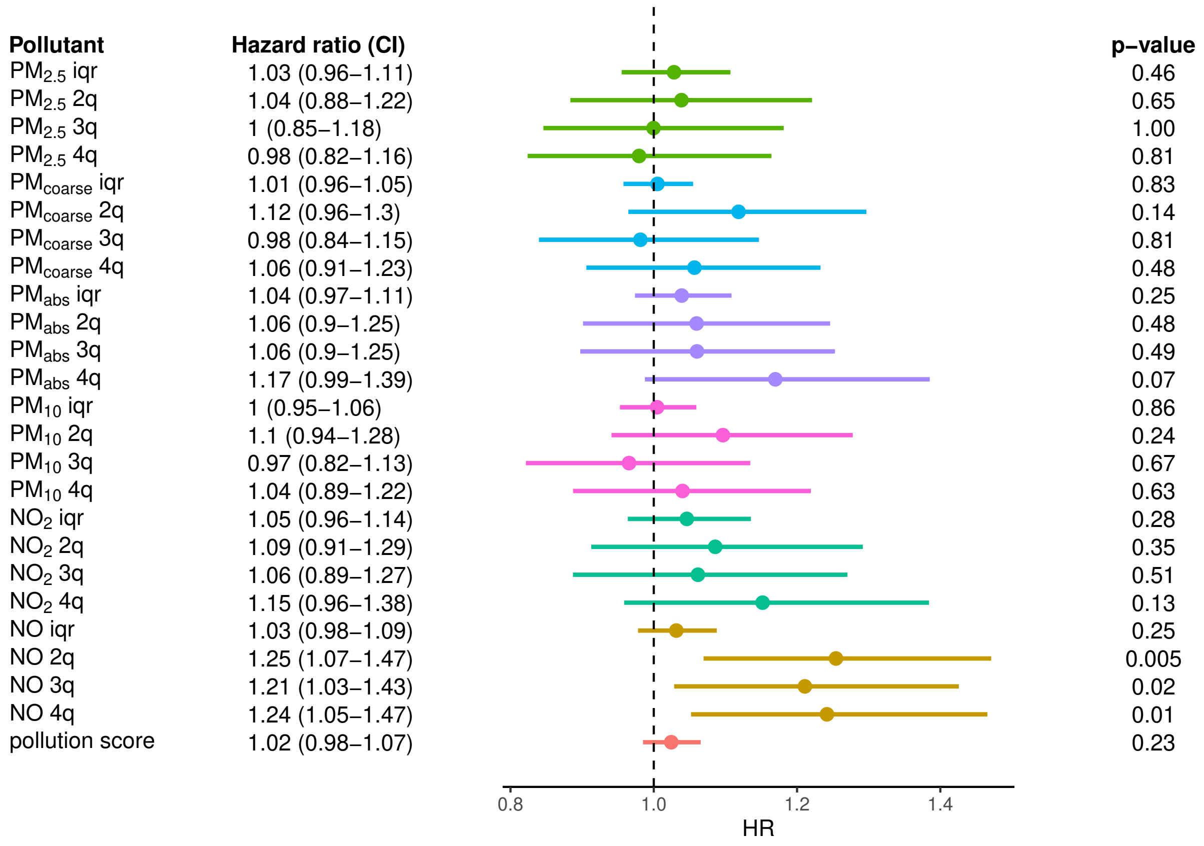

Figure 3: Effect of air pollution on VAD. From left to right, for each air pollution exposure we show its associated hazard ratio and confidence interval, the forest plot and p-value of the effect estimate (Wald test); ‘iqr’ and ‘n^{th}q’ refer to the continuous (scale by IQR) and discrete exposures (n^{th} quartile), respectively. The models were adjusted for age, sex, ethnicity, educational attainment, income, and population density.

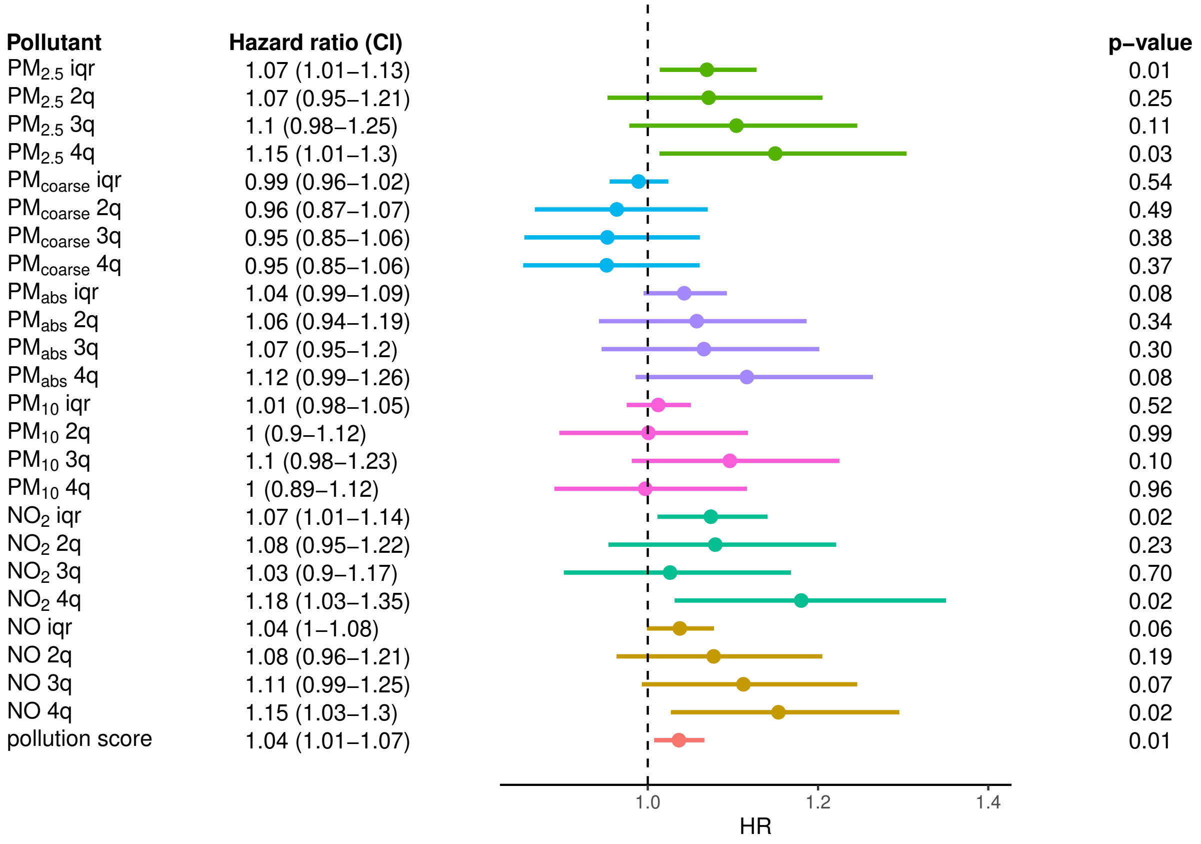

Figure 4: Effect of air pollution on AD. From left to right, we show the pollution exposure with the associated hazard ratio and confidence interval, the forest plot and p-value of the effect estimate (Wald test); 'iqr' and 'n^{th}q' refer to the continuous (scale by IQR) and discrete exposures (n^{th} quartile), respectively. The models were adjusted for age, sex, ethnicity, educational attainment, income, population density, and IMD.

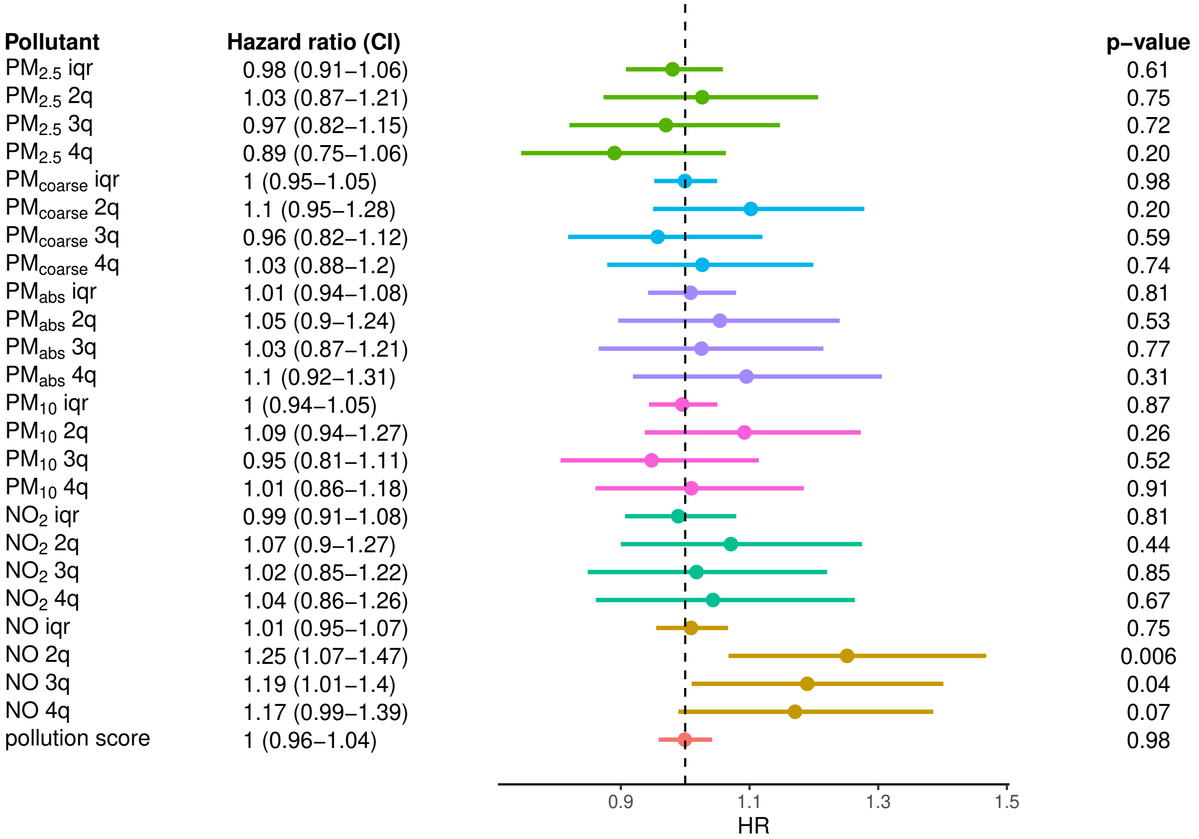

Figure 5: Effect of air pollution on VAD. From left to right, we show the pollution exposure with the associated hazard ratio and confidence interval, the forest plot and p-value of the effect estimate (Wald test); 'iqr' and 'n^{th}q' refer to the continuous (scale by IQR) and discrete exposures (n^{th} quartile), respectively. The models were adjusted for age, sex, ethnicity, educational attainment, income, population density, and IMD.

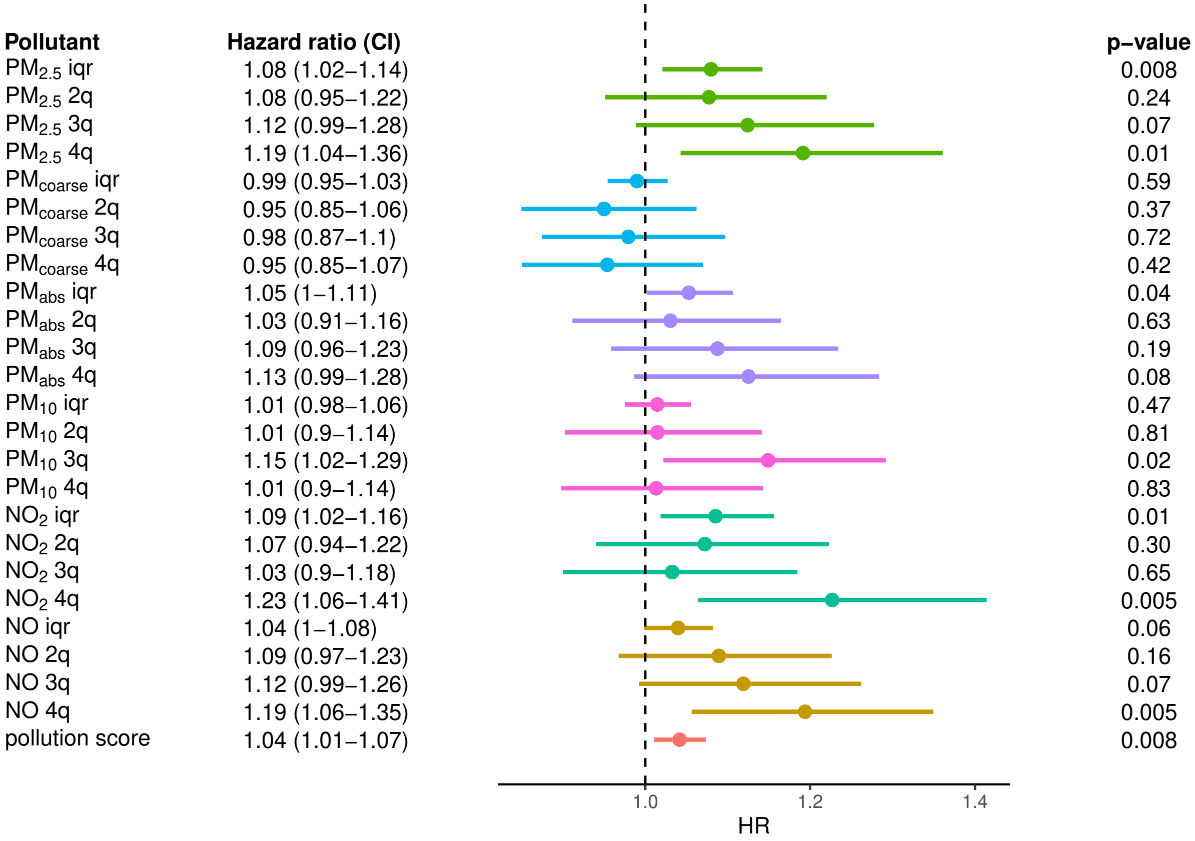

Figure 6: Effect of air pollution on AD. From left to right, we show the pollution exposure with the associated hazard ratio and confidence interval, the forest plot and p-value of the effect estimate (Wald test); 'iqr' and 'n^{th}q' refer to the continuous (scale by IQR) and discrete exposures (n^{th} quartile), respectively. The models were adjusted for age, sex, ethnicity, educational attainment, income, population density, and IMD. Additionally, here we excluded participants who lived less than 5 years (instead of 1 year) at baseline address.

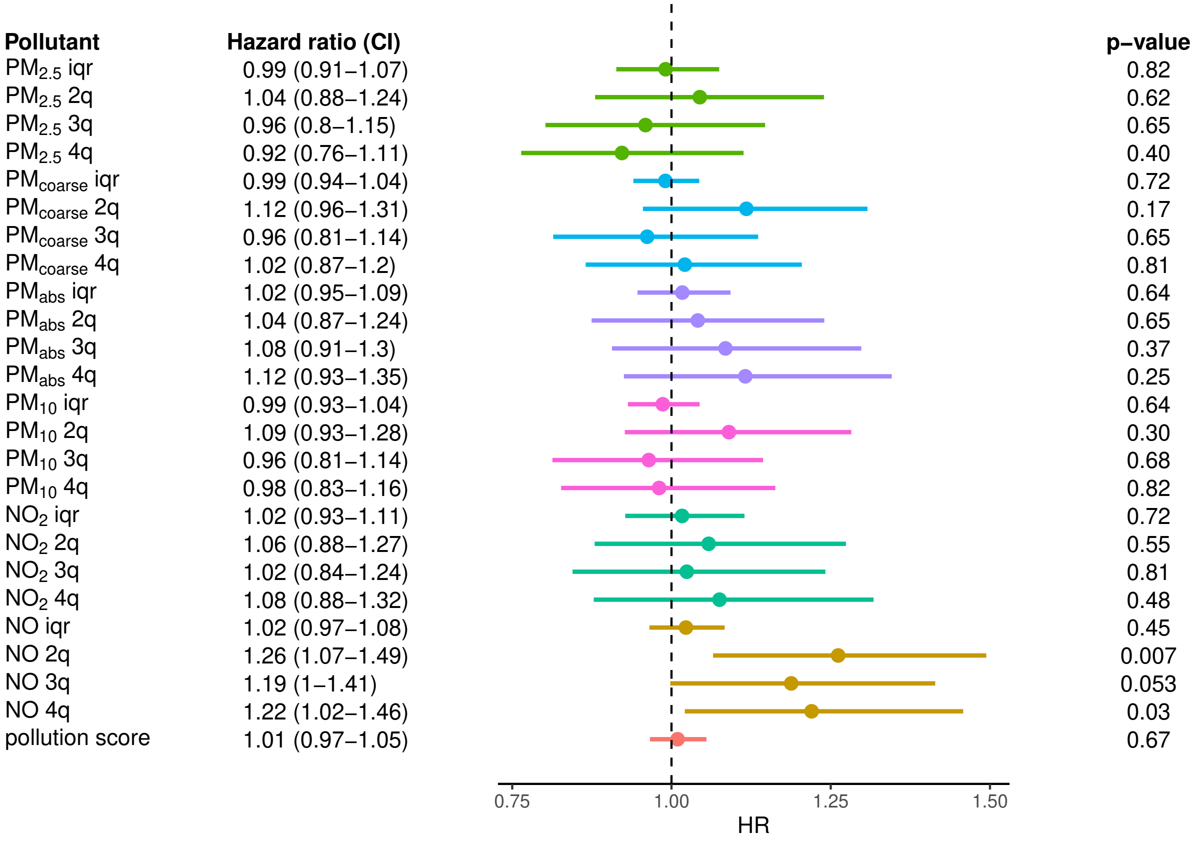

Figure 7: Effect of air pollution on VAD. From left to right, we show the pollution exposure with the associated hazard ratio and confidence interval, the forest plot and p-value of the effect estimate (Wald test); ‘iqr’ and ‘n^{th}q’ refer to the continuous (scale by IQR) and discrete exposures (n^{th} quartile), respectively. The models were adjusted for age, sex, ethnicity, educational attainment, income, population density, and IMD. Additionally, here we excluded participants who lived less than 5 years (instead of 1 year) at baseline address.

4 Mixture exposure analysis

The mixture model used for this analysis is:

$$\lambda(t|X) = \lambda_0 exp(\sum_{j=1}^{m} \beta_j X_j + \mathbf{X_c}'\boldsymbol{\beta_c}) \tag{1}$$

where m is the number of pollutants included in the mixture, β_j is the coefficient for the jth pollutant, encoded as quartile, $\mathbf{X_c}$ is the covariate vector with associated coefficient vector $\boldsymbol{\beta_c}$. This mixture model is used to estimate the mixture coefficient $\psi = \sum \beta_j$, which can be interpreted as the effect of increasing by one quartile all components of the mixture simultaneously. Notably, despite the estimation of the β's associated with the mixture components can be unstable due to multicollinearity, their summed effect can be reliably estimated. For this analysis we use the package qgcomp [9] (v. 2.18.7), and in particular the function qgcomp.cox.noboot, which returns the mixture hazard ratio conditional on the covariates. The results of this analysis are shown in Table 1-3 for models with either all pollutants included in the mixture or excluding PM_{10} and PM_{coarse}, and with all-cause dementia, AD, and VAD as an outcome, respectively.

	Estimate	Std. Error	Lower CI	Upper CI	p-value
all	1.041	1.019	1.003	1.080	0.034
restricted	1.052	1.018	1.017	1.089	0.003

Table 1: Conditional effects ψ, with associated standard error and p-value, of the exposure to a mixture of air pollutants on all-cause dementia hazard. Mixture could include all pollutants or exclude PM_{10} and PM_{coarse} ("restricted").

	Estimate	Std. Error	Lower CI	Upper CI	p-value
all	1.064	1.029	1.006	1.124	0.029
restricted	1.084	1.027	1.030	1.142	0.002

Table 2: Conditional effects ψ, with associated standard error and p-value, of the exposure to a mixture of air pollutants on AD hazard. Mixture could include all pollutants or exclude PM_{10} and PM_{coarse} ("restricted").

	Estimate	Std. Error	Lower CI	Upper CI	p-value
all	1.023	1.041	0.945	1.106	0.579
restricted	1.036	1.038	0.963	1.114	0.344

Table 3: Conditional effects ψ, with associated standard error and p-value, of the exposure to a mixture of air pollutants on VAD hazard. Mixture could include all pollutants or exclude PM_{10} and PM_{coarse} ("restricted").

5 Variable selection analysis

We used three methods in order to identify the most relevant air pollutants for dementia risk. First, we used Cox regression with a Lasso (L1) penalisation in models including all air pollutants. This penalisation constraints the coefficients towards 0, while setting a subset of them to 0, enforcing sparsity. We ran the analysis adjusting for IMD, in addition to baseline covariates, and by using both 1 and 5 years as a threshold for time at residential address. We implemented 10-fold cross-validated regularised Cox regression with L1 penalty and C-index as a loss function using the package glmnet. The L1 penalty was only applied to coefficients associated to air pollutants, thus it did not affect estimation of covariate coefficients. Second, we used elastic net regularisation in place of Lasso penalisation using the same model, package and cross-validation setup. Lasso regression can in fact be unstable with highly correlated variables and randomly select one element in a group of strongly correlated variables with similar effects on the outcome (up to sign). Elastic net, which uses as penalty a mixture of L1 and L2 (Ridge) norms, resolves this problem by allowing the selection of groups of collinear variables with comparable effects on the outcome [10]. We used a linear mixture of L1 and L2 penalty with weights 0.95 and 0.5 respectively, as this allows to retain the variable selection behaviour driven by the L1 penalty while avoiding random selection. It is important to notice, however, that Elastic net is particularly useful in settings where correlations among variables highlight shared causal pathways, like in a genetic context; however, in our setting correlations among pollutants – likely driven by similar behavioural patterns of exposure – may bare moderate to little significance in terms of shared causal neurotoxic pathways. For this reason, as a third method we used weighted quantile sum (WQS) regression, which allows us to estimate the effects β_m of a mixture of pollutants on the outcome, as well as the individual contribution w_j of each pollutant to the mixture, without encouraging grouping [11]. The estimation of individual pollutant weights is possible in presence of multicollinearity because WQS imposes constraints on the weights, and in particular $w_j \geq 0$ and $\sum_{j=1}^{m} = 1$, where m is equal to the number of pollutant components in the mixture. We used the gWQS package (v. 3.0.5), which does not allow direct implementation of this method in a survival analysis context; instead, we used WQS logistic regression with incidence of dementia after baseline as an outcome. In more details, using maximum likelihood estimation, the weight associated to the jth pollutant was given by $w_j = \frac{1}{B}\sum_{b=1}^{B} w_{j(b)} f(\beta_{(b)})$, where $B = 50$ is the number of bootstrap samples drawn from the entire dataset, $w_{j(b)}$ is the weight for pollutant j^{th} estimated for a given bootstrap sample, $\beta_{(b)}$ is the estimated mixture effect for the same bootstrap sample, and f is a linear function constrained to sum to 1 across bootstraps. For both regression with elastic net regularisation and WQS regression we only used 5 years as threshold for time at residential address. Results from this analysis are shown in Table 4.

	lasso $\geq$ 1 year	lasso $\geq$ 5 year lasso	elastic $\geq$ 5 year	wqs $\geq$ 5 year
$PM_{2.5}$	1.000	1.000	1.000	0.261
PM_{coarse}	1.000	1.000	1.000	0.007
PM_{abs}	1.000	1.000	1.000	0.058
PM_{10}	1.000	1.000	1.000	0.004
NO_2	1.001	1.002	1.002	0.359
NO	1.000	1.000	1.000	0.311

Table 4: Variable selection for air pollutants. The first three columns of the table include pollutant-specific coefficients (hazard ratios) for models either excluding participants with less than 1 year or less than 5 years at baseline residence using lasso or elastic net regularisation. The strength of the penalisation is a free parameter, which here was chosen based on 10-fold cross-validated C index, an evaluation metrics for survival models. The fourth column shows the weights assigned to each pollutant in constructing an exposure mixture in a WQS regression model, where higher weights indicate stronger contribution to the mixture. All models were adjusted for age, sex, ethnicity, educational attainment, income, population density, and IMD.

6 Recruitment centre random effects

Random effect models included a centre-specific intercept and slope for the effect of air pollution on dementia. We ran the analysis adjusting for IMD, in addition to baseline covariates, and by using both 1 and 5 years as a threshold for time at residential address; for each model, after exclusion criteria were applied, the number of recruitment centres were 18 and 17, respectively. Figure 8 and table 5 display the distribution and standard deviations of the slopes, respectively.

	sd	sd (HR scale)	HR mean
$PM_{2.5} \geq 1$ year	0.038	1.0387	1.005
$PM_{2.5} \geq 5$ years	0.059	1.0608	1.013
$PM_{coarse} \geq 1$ year	0.006	1.0060	1.000
$PM_{coarse} \geq 5$ years	0.008	1.0080	1.000
$PM_{abs} \geq 1$ year	0.041	1.0419	1.002
$PM_{abs} \geq 5$ years	0.048	1.0492	1.006
$PM_{10} \geq 1$ year	0.005	1.0050	1.000
$PM_{10} \geq 5$ years	0.003	1.0030	1.000
$NO_2 \geq 1$ year	0.063	1.0650	1.010
$NO_2 \geq 5$ years	0.076	1.0790	1.019
$NO \geq 1$ year	0.041	1.0419	1.005
$NO \geq 5$ years	0.042	1.0429	1.006
pollution score ≥ 1 year	0.029	1.0294	1.003
pollution score ≥ 5 years	0.034	1.0346	1.007

Table 5: Distribution of centre-specific effect measures. In the table we show standard deviation of the pollution-specific random effects (left) and the mean of the hazard ratios over recruitment centres (right). In addition, we show the hazard ratio associated with an increase of the pollution coefficient by 1 standard deviation above 0 (middle). We display the results when excluding participants who lived either less than 1 or 5 years at baseline address.

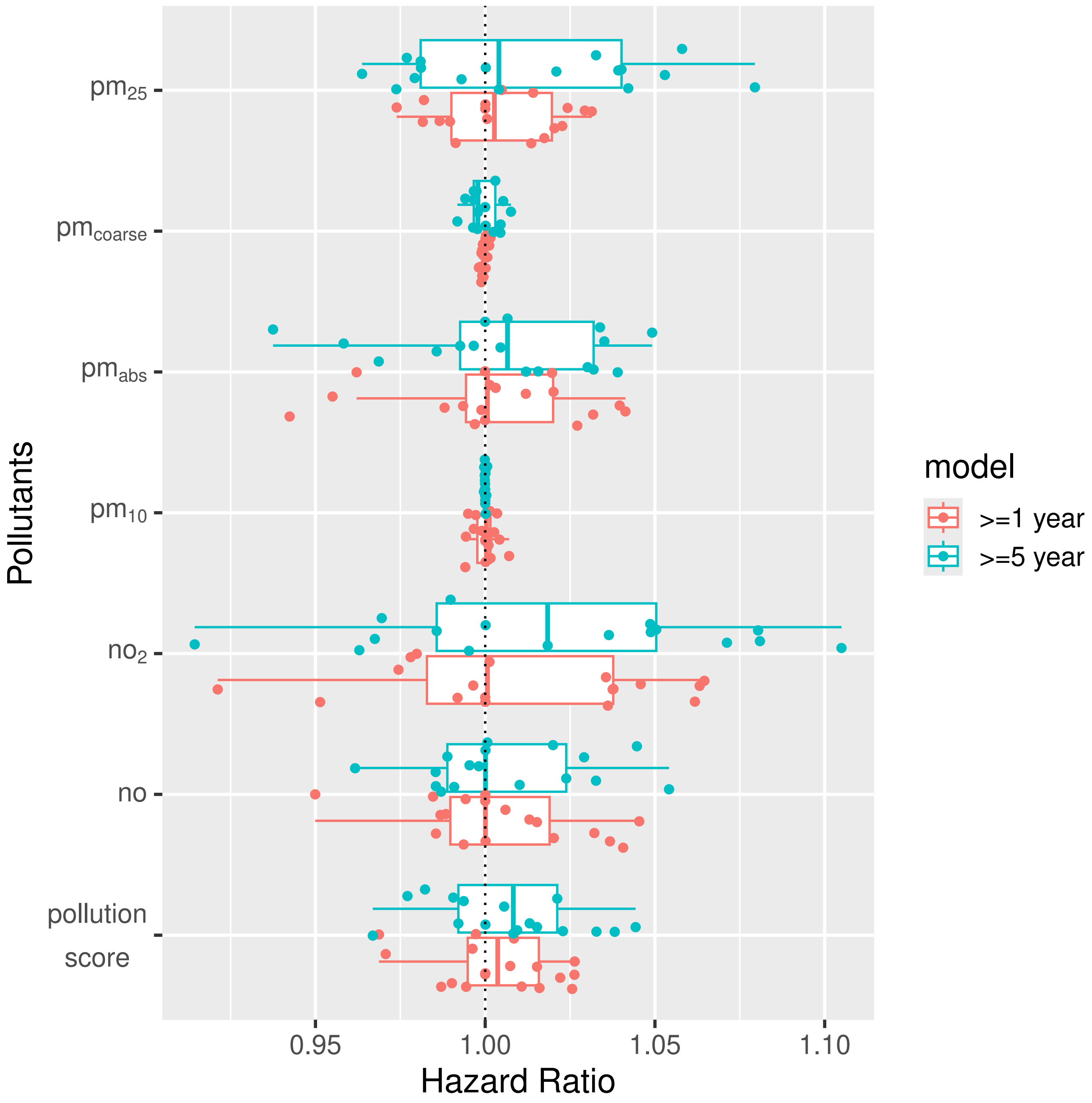

Figure 8: Pollutant-specific effects on dementia across recruitment centres. The boxplots include the 1st, 2nd and 3rd quartiles, plus the minimum and maximum values within 1.5*IQR; superimposed on the boxplots are the centre-specific estimates (dots). For each air pollutant we ran two analyses, excluding participants who lived either less than 1 year (red) or less than 5 years (cyan) at baseline residence; for the second analysis there was one less recruitment centre, for a total of 17 (Swansea was dropped due to lack of participants). The models were adjusted for age, sex, ethnicity, educational attainment, income, population density, and IMD.

7 Positive control analysis

We used Cox regression to assess whether air pollution affects time to COPD occurrence. Figure 9, 10 and 11 show the results for the pollution score and single-pollutant models respectively without IMD, with IMD as a covariate and using a 5-year cutoff for time at residential address.

Figure 12 shows the distribution of changes in HR across air pollutants when using 5 vs 1 year cutoff for time at baseline address, for all-cause dementia and COPD as an outcome.

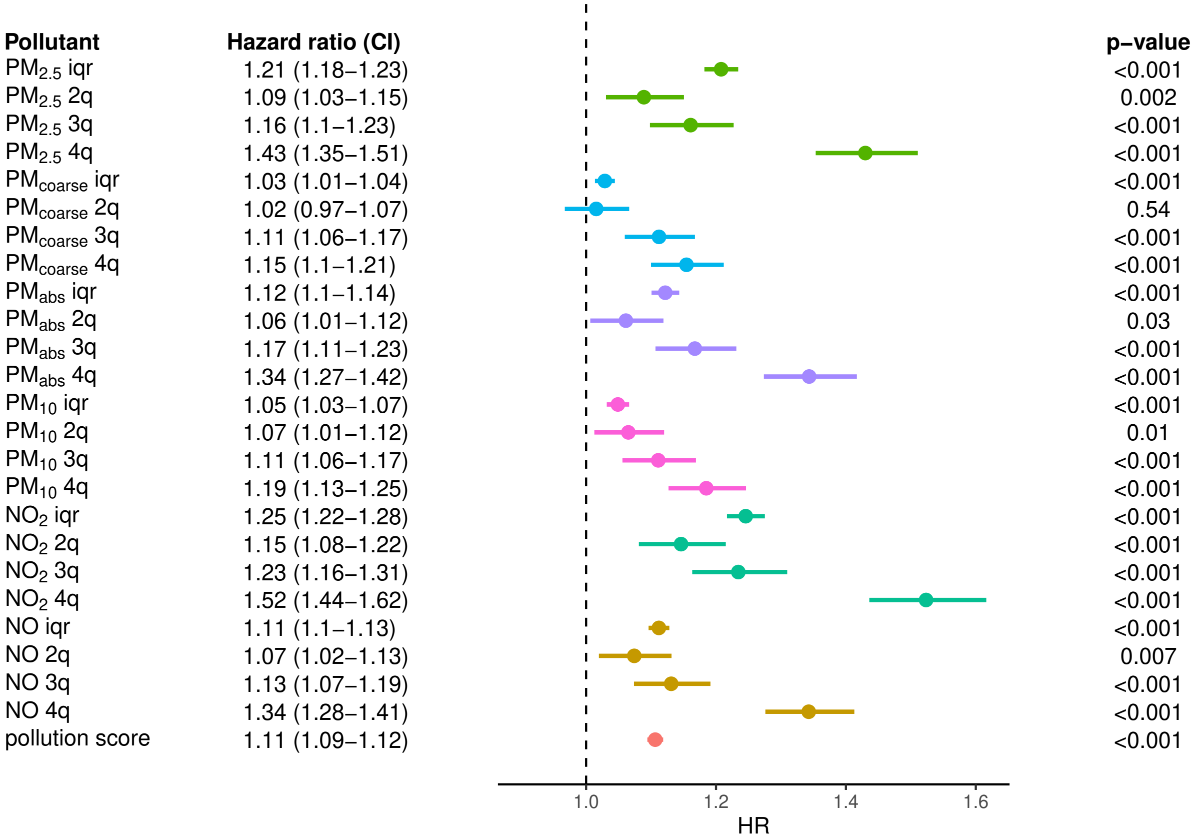

Figure 9: Effect of air pollution on COPD. From left to right, for each air pollution exposure we show its associated hazard ratio and confidence interval, the forest plot and p-value of the effect estimate (Wald test); 'iqr' and 'n^{th}q' refer to the continuous (scale by IQR) and discrete exposures (n^{th} quartile), respectively. The models were adjusted for age, sex, ethnicity, educational attainment, income, and population density.

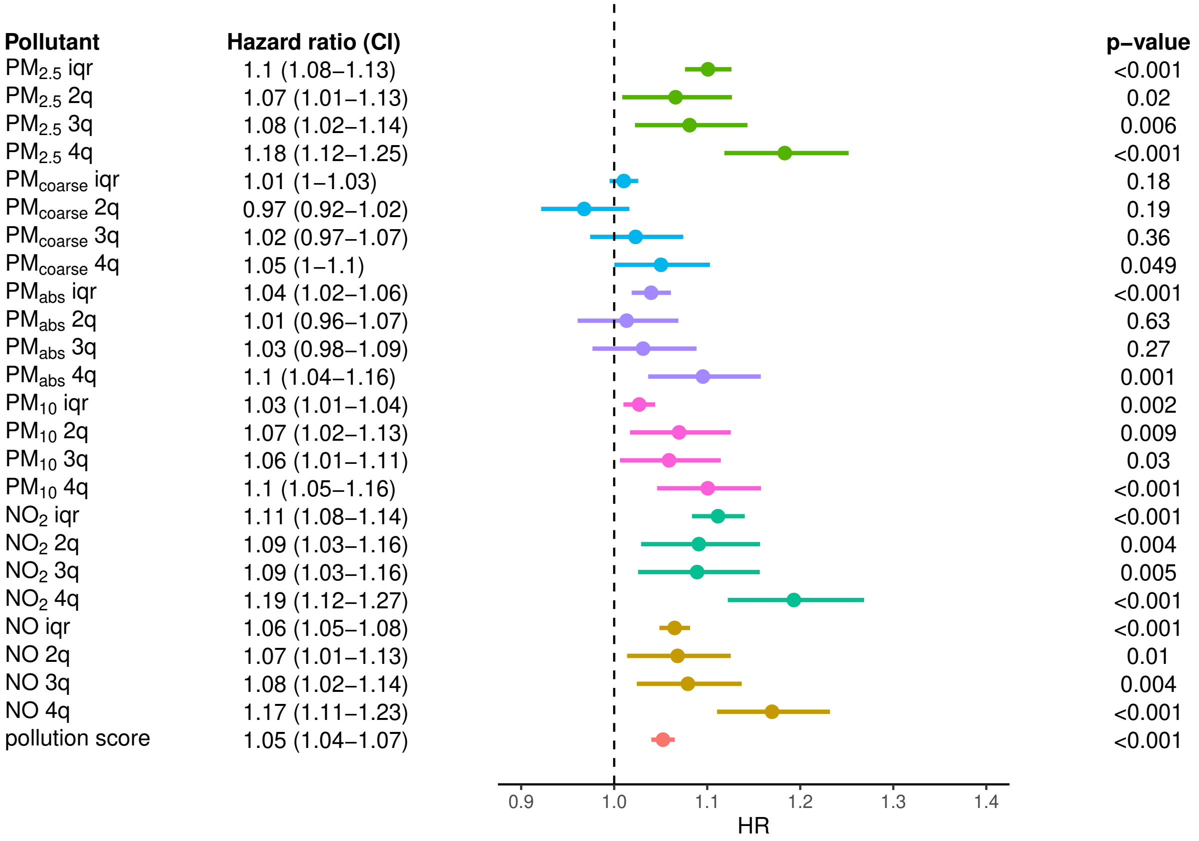

Figure 10: Effect of air pollution on COPD. From left to right, for each air pollution exposure we show its associated hazard ratio and confidence interval, the forest plot and p-value of the effect estimate (Wald test); 'iqr' and 'n^{th}q' refer to the continuous (scale by IQR) and discrete exposures (n^{th} quartile), respectively. The models were adjusted for age, sex, ethnicity, educational attainment, income, population density, and IMD.

Pollutant	Hazard ratio (CI)	p–value
$PM_{2.5}$ iqr	1.1 (1.07–1.13)	<0.001
$PM_{2.5}$ 2q	1.05 (0.99–1.12)	0.08
$PM_{2.5}$ 3q	1.07 (1–1.13)	0.04
$PM_{2.5}$ 4q	1.17 (1.1–1.25)	<0.001
PM_{coarse} iqr	1.02 (1–1.03)	0.06
PM_{coarse} 2q	0.97 (0.92–1.02)	0.21
PM_{coarse} 3q	1.01 (0.96–1.06)	0.73
PM_{coarse} 4q	1.06 (1.01–1.12)	0.02
PM_{abs} iqr	1.04 (1.02–1.07)	<0.001
PM_{abs} 2q	1.02 (0.96–1.08)	0.47
PM_{abs} 3q	1.03 (0.97–1.1)	0.28
PM_{abs} 4q	1.11 (1.04–1.18)	<0.001
PM_{10} iqr	1.03 (1.01–1.05)	<0.001
PM_{10} 2q	1.07 (1.01–1.13)	0.02
PM_{10} 3q	1.05 (0.99–1.11)	0.08
PM_{10} 4q	1.11 (1.05–1.17)	<0.001
NO_2 iqr	1.12 (1.09–1.15)	<0.001
NO_2 2q	1.09 (1.02–1.16)	0.007
NO_2 3q	1.08 (1.01–1.15)	0.02
NO_2 4q	1.21 (1.13–1.29)	<0.001
NO iqr	1.07 (1.05–1.09)	<0.001
NO 2q	1.06 (1–1.12)	0.03
NO 3q	1.06 (1–1.12)	0.04
NO 4q	1.18 (1.12–1.25)	<0.001
pollution score	1.06 (1.04–1.07)	<0.001

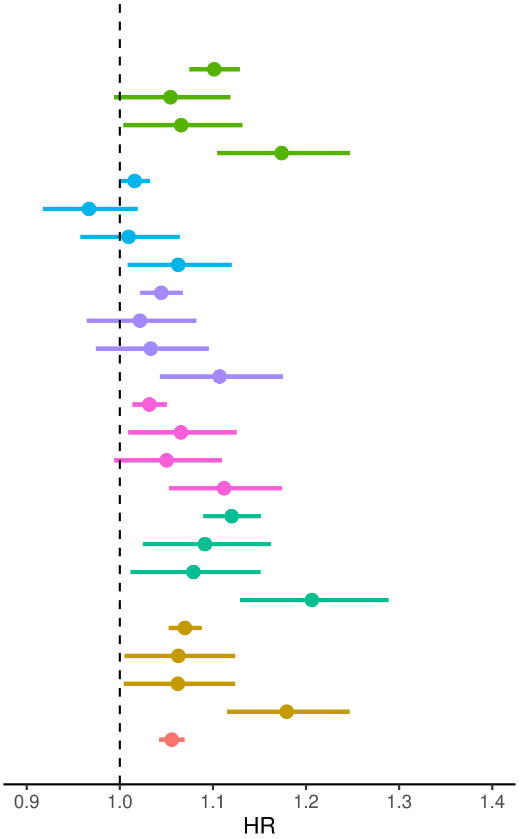

Figure 11: Effect of air pollution on COPD. From left to right, we show the pollution exposure with the associated hazard ratio and confidence interval, the forest plot and p-value of the effect estimate (Wald test); 'iqr' and 'n^{th}q' refer to the continuous (scale by IQR) and discrete exposures (n^{th} quartile), respectively. The models were adjusted for age, sex, ethnicity, educational attainment, income, population density, and IMD. Additionally, here we excluded participants who lived less than 5 years (instead of 1 year) at baseline address.

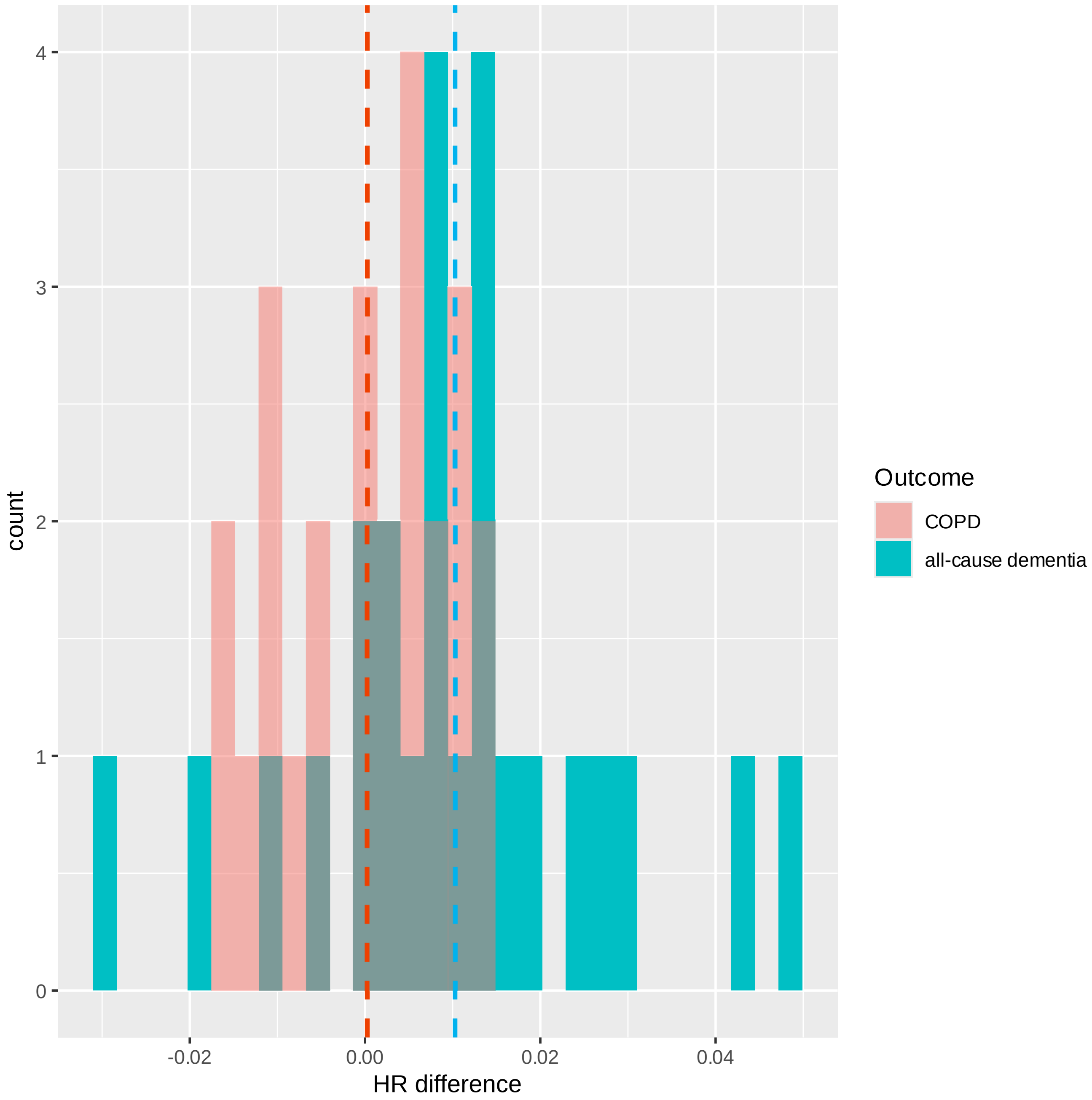

Figure 12: Difference in HR for 5- vs 1-year cutoff. The two histograms show the distribution of differences in hazard ratio (HR) when using 5- vs 1-year cutoff for time lived at baseline address, for all-cause dementia (cyan) and COPD (red) as an outcome. Positive values indicate a higher risk of dementia detected in the analyses using 5-year cutoff. Vertical dashed lines indicate the mean of the distributions. Comparing the two distributions, it appears that there is a larger increase in HR for dementia compared to COPD (Wilcoxon paired test, T=73, p=0.015)

8 Negative control analysis

We used ordinal logistic regression to assess whether we could detect an association between air pollution exposure and oily fish intake frequency after adjusting for confounders. Oily fish consumption was coded from higher to lower intake frequency, so that increased odds ratios indicate lower probability of fish intake. Table 6 shows the results for the pollution score and single-pollutant models, with oily fish intake frequency as an ordinal outcome, and exposure encoded continuously, scaled by inter-quartile range; we did not considered air pollution quartiles as an exposure for this analysis. For the pollution score model, the exposure was the first principal component capturing variability in exposure to $PM_{2.5}$, PM_{abs}, NO_2 and NO.

	model	OR	L95	U95
$PM_{2.5}$	IMD anudjusted	0.99	0.98	1.00
	IMD adjusted	0.97	0.97	0.98
PM_{coarse}	IMD anudjusted	0.99	0.98	0.99
	IMD udjusted	0.99	0.98	0.99
PM_{abs}	IMD anudjusted	0.97	0.96	0.98
	IMD adjusted	0.95	0.95	0.96
PM_{10}	IMD anudjusted	0.99	0.99	1.00
	IMD adjusted	0.99	0.98	1.00
NO_2	IMD anudjusted	0.98	0.97	0.99
	IMD adjusted	0.96	0.95	0.97
NO	IMD anudjusted	0.99	0.99	1.00
	IMD adjusted	0.99	0.98	0.99
pollution score	IMD anudjusted	0.99	0.99	0.99
	IMD adjusted	0.98	0.98	0.98

Table 6: Effect of air pollution of oily fish consumption. Odds ratios lower than one indicate a decrease in probability of consuming less fish, or otherwise an increase in probability of consuming more fish. The models were adjusted for age, sex, ethnicity, educational attainment, income, and population density; additionally, the models could be adjusted for index of multiple deprivation (IMD). L95 and U95 correspond to the lower and upper limit of the 95% confidence interval.

9 Control for noise pollution

Figure 13 displays the result from Cox regressions using pollution score and single-pollutant models that included noise pollution as an additional covariate.

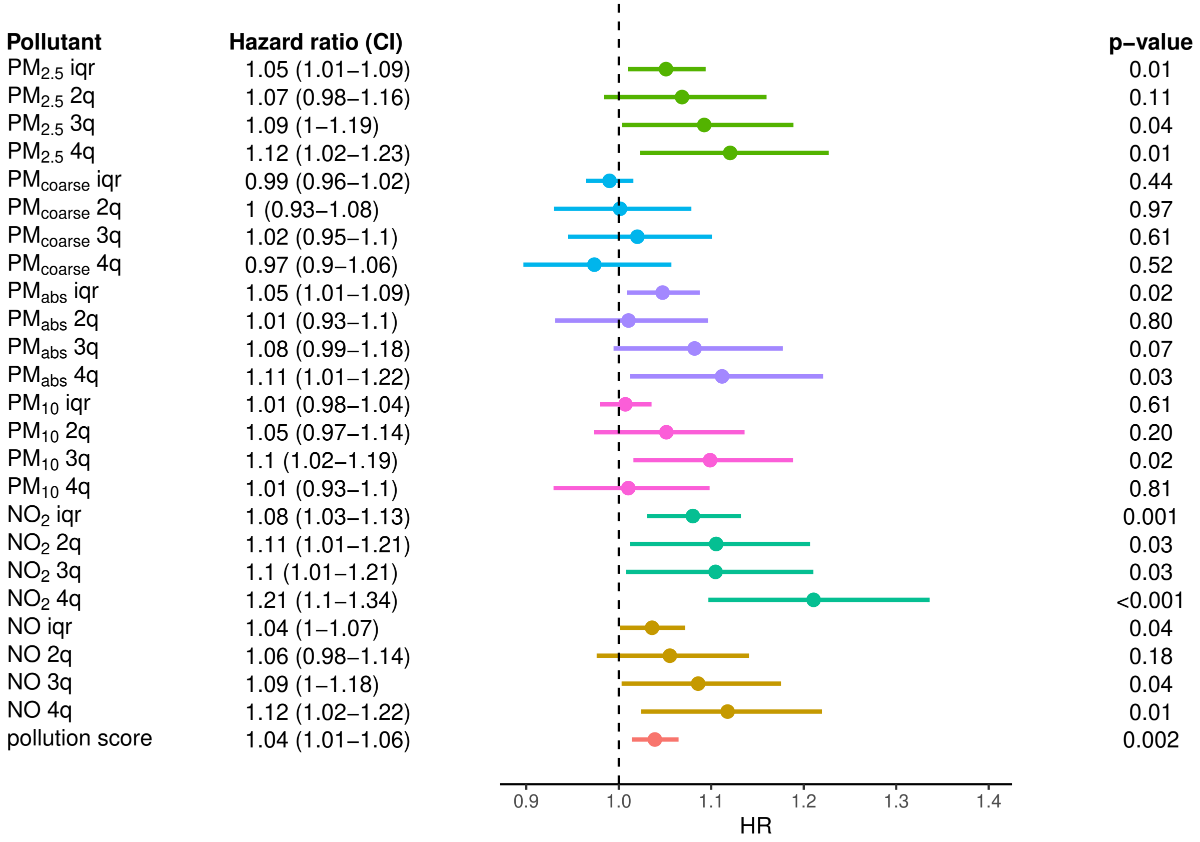

Figure 13: Effect of air pollutants of risk of all-cause dementia when controlling for noise pollution. From left to right, for each air pollution exposure we show its associated hazard ratio and confidence interval, the forest plot and p-value of the effect estimate (Wald test). The models were adjusted for age, sex, ethnicity, educational attainment, income, population density, IMD and noise pollution. Additionally, here we excluded participants who lived less than 5 years (instead of 1 year) at baseline address.

10 Effect modification by deprivation level

We assessed whether the effect of air pollution on dementia is modified by deprivation, both on the multiplicative and additive scale. In the latter case, we used the relative excess risk due to interaction (RERI), which gives the direction of the additive modification. Tables 7-20 report the estimated effect modification on the multiplicative and additive scale, together with the hazard ratio for each combination of exposure and deprivation: in particular, HR10 is the HR for participants with air pollution exposure of 1 iqr above the median and assigned the 1st quartile of IMD, HR01 is the HR for participants assigned the 4th quartile of IMD and with air pollution exposure equal to the median, and HR11 is the HR for participants both with air pollution exposure of 1 IQR above the medial and assigned the 4th quartile of IMD. The interaction on the multiplicative scale is given by $\exp^{\beta_{11}}$=HR11/(HR01*HR10), where β_{11} is the interaction coefficient in the linear model of the Cox regression; the RERI is given by HR11-HR10-HR01-1. We ran the analysis both when excluding participants who lived less that 1 year and when excluding participants who less than 5 years at baseline address.

	Measures	Estimates	L95	U95	p-value
1	HR00	1.000	NA	NA	NA
2	HR01	1.264	1.167	1.370	0.000
3	HR10	1.046	0.960	1.140	0.305
4	HR11	1.304	1.202	1.415	0.000
7	Multiplicative scale	0.986	0.892	1.091	0.786
8	RERI	-0.006	-0.119	0.107	0.542

Table 7: Effect modification for $PM_{2.5}$. L95 and U95 correspond to the lower and upper limit of the 95% confidence interval. The model was adjusted for adjusted for age, sex, ethnicity, educational attainment, income, population density, and IMD. Hazard ratio (HR), relative excess risk due to interaction (RERI), not a number (NA).

	Measures	Estimates	L95	U95	p-value
1	HR00	1.000	NA	NA	NA
2	HR01	1.249	1.146	1.360	0.000
3	HR10	1.066	0.973	1.168	0.171
4	HR11	1.313	1.205	1.431	0.000
7	Multiplicative scale	0.986	0.886	1.098	0.801
8	RERI	-0.002	-0.124	0.120	0.511

Table 8: Effect modification for $PM_{2.5}$. L95 and U95 correspond to the lower and upper limit of the 95% confidence interval. The model was adjusted for adjusted for age, sex, ethnicity, educational attainment, income, population density, and IMD. Additionally, here we excluded participants who lived less than 5 years (instead of 1 year) at baseline address. Hazard ratio (HR), relative excess risk due to interaction (RERI), not a number (NA).

	Measures	Estimates	L95	U95	p-value
1	HR00	1.000	NA	NA	NA
2	HR01	1.286	1.188	1.393	0.000
3	HR10	1.003	0.922	1.091	0.941
4	HR11	1.298	1.199	1.405	0.000
7	Multiplicative scale	1.006	0.913	1.108	0.907
8	RERI	0.008	-0.097	0.113	0.438

Table 9: Effect modification for PM_{abs}. L95 and U95 correspond to the lower and upper limit of the 95% confidence interval. The model was adjusted for adjusted for age, sex, ethnicity, educational attainment, income, population density, and IMD. Hazard ratio (HR), relative excess risk due to interaction (RERI), not a number (NA).

	Measures	Estimates	L95	U95	p-value
1	HR00	1.000	NA	NA	NA
2	HR01	1.265	1.162	1.378	0.000
3	HR10	1.031	0.944	1.126	0.496
4	HR11	1.305	1.200	1.419	0.000
7	Multiplicative scale	1.000	0.903	1.107	0.999
8	RERI	0.008	-0.105	0.121	0.443

Table 10: Effect modification for PM_{abs}. L95 and U95 correspond to the lower and upper limit of the 95% confidence interval. The model was adjusted for adjusted for age, sex, ethnicity, educational attainment, income, population density, and IMD. Additionally, here we excluded participants who lived less than 5 years (instead of 1 year) at baseline address. Hazard ratio (HR), relative excess risk due to interaction (RERI), not a number (NA).

	Measures	Estimates	L95	U95	p-value
1	HR00	1.000	NA	NA	NA
2	HR01	1.307	1.209	1.412	0.000
3	HR10	0.984	0.937	1.032	0.504
4	HR11	1.265	1.169	1.368	0.000
7	Multiplicative scale	0.984	0.922	1.051	0.631
8	RERI	-0.025	-0.100	0.049	0.749

Table 11: Effect modification for PM_{coarse}. L95 and U95 correspond to the lower and upper limit of the 95% confidence interval. The model was adjusted for adjusted for age, sex, ethnicity, educational attainment, income, population density, and IMD. Hazard ratio (HR), relative excess risk due to interaction (RERI), not a number (NA).

	Measures	Estimates	L95	U95	p-value
1	HR00	1.000	NA	NA	NA
2	HR01	1.304	1.200	1.416	0.000
3	HR10	0.993	0.944	1.045	0.783
4	HR11	1.273	1.171	1.384	0.000
7	Multiplicative scale	0.983	0.918	1.054	0.635
8	RERI	-0.024	-0.102	0.055	0.721

Table 12: Effect modification for PM_{coarse}. L95 and U95 correspond to the lower and upper limit of the 95% confidence interval. The model was adjusted for adjusted for age, sex, ethnicity, educational attainment, income, population density, and IMD. Additionally, here we excluded participants who lived less than 5 years (instead of 1 year) at baseline address. Hazard ratio (HR), relative excess risk due to interaction (RERI), not a number (NA).

	Measures	Estimates	L95	U95	p-value
1	HR00	1.000	NA	NA	NA
2	HR01	1.297	1.203	1.399	0.000
3	HR10	1.012	0.960	1.065	0.665
4	HR11	1.279	1.179	1.388	0.000
7	Multiplicative scale	0.975	0.910	1.045	0.469
8	RERI	-0.030	-0.109	0.049	0.770

Table 13: Effect modification for PM_{10}. L95 and U95 correspond to the lower and upper limit of the 95% confidence interval. The model was adjusted for adjusted for age, sex, ethnicity, educational attainment, income, population density, and IMD. Hazard ratio (HR), relative excess risk due to interaction (RERI), not a number (NA).

	Measures	Estimates	L95	U95	p-value
1	HR00	1.000	NA	NA	NA
2	HR01	1.292	1.192	1.401	0.000
3	HR10	1.028	0.973	1.086	0.321
4	HR11	1.286	1.180	1.402	0.000
7	Multiplicative scale	0.968	0.899	1.042	0.383
8	RERI	-0.035	-0.119	0.050	0.788

Table 14: Effect modification for PM_{10}. L95 and U95 correspond to the lower and upper limit of the 95% confidence interval. The model was adjusted for adjusted for age, sex, ethnicity, educational attainment, income, population density, and IMD. Additionally, here we excluded participants who lived less than 5 years (instead of 1 year) at baseline address. Hazard ratio (HR), relative excess risk due to interaction (RERI), not a number (NA).

	Measures	Estimates	L95	U95	p-value
1	HR00	1.000	NA	NA	NA
2	HR01	1.264	1.163	1.373	0.000
3	HR10	1.034	0.939	1.139	0.496
4	HR11	1.315	1.207	1.432	0.000
7	Multiplicative scale	1.006	0.899	1.126	0.915
8	RERI	0.017	-0.109	0.143	0.396

Table 15: Effect modification for NO_2. L95 and U95 correspond to the lower and upper limit of the 95% confidence interval. The model was adjusted for adjusted for age, sex, ethnicity, educational attainment, income, population density, and IMD. Hazard ratio (HR), relative excess risk due to interaction (RERI), not a number (NA).

	Measures	Estimates	L95	U95	p-value
1	HR00	1.000	NA	NA	NA
2	HR01	1.243	1.138	1.358	0.000
3	HR10	1.058	0.955	1.172	0.284
4	HR11	1.327	1.213	1.452	0.000
7	Multiplicative scale	1.009	0.896	1.137	0.880
8	RERI	0.026	-0.110	0.163	0.354

Table 16: Effect modification for NO_2. L95 and U95 correspond to the lower and upper limit of the 95% confidence interval. The model was adjusted for adjusted for age, sex, ethnicity, educational attainment, income, population density, and IMD. Additionally, here we excluded participants who lived less than 5 years (instead of 1 year) at baseline address. Hazard ratio (HR), relative excess risk due to interaction (RERI), not a number (NA).

	Measures	Estimates	L95	U95	p-value
1	HR00	1.000	NA	NA	NA
2	HR01	1.275	1.180	1.378	0.000
3	HR10	1.017	0.947	1.093	0.635
4	HR11	1.304	1.207	1.408	0.000
7	Multiplicative scale	1.005	0.926	1.090	0.906
8	RERI	0.011	-0.078	0.100	0.402

Table 17: Effect modification for NO. L95 and U95 correspond to the lower and upper limit of the 95% confidence interval. The model was adjusted for adjusted for age, sex, ethnicity, educational attainment, income, population density, and IMD. Hazard ratio (HR), relative excess risk due to interaction (RERI), not a number (NA).

	Measures	Estimates	L95	U95	p-value
1	HR00	1.000	NA	NA	NA
2	HR01	1.268	1.168	1.377	0.000
3	HR10	1.048	0.972	1.129	0.223
4	HR11	1.301	1.199	1.412	0.000
7	Multiplicative scale	0.980	0.899	1.067	0.637
8	RERI	-0.014	-0.110	0.081	0.616

Table 18: Effect modification for NO. L95 and U95 correspond to the lower and upper limit of the 95% confidence interval. The model was adjusted for adjusted for age, sex, ethnicity, educational attainment, income, population density, and IMD. Additionally, here we excluded participants who lived less than 5 years (instead of 1 year) at baseline address. Hazard ratio (HR), relative excess risk due to interaction (RERI), not a number (NA).

	Measures	Estimates	L95	U95	p-value
1	HR00	1.000	NA	NA	NA
2	HR01	1.266	1.167	1.373	0.000
3	HR10	1.017	0.968	1.069	0.497
4	HR11	1.287	1.193	1.390	0.000
7	Multiplicative scale	0.999	0.944	1.058	0.985
8	RERI	0.004	-0.058	0.066	0.451

Table 19: Effect modification for pollution score. L95 and U95 correspond to the lower and upper limit of the 95% confidence interval. The model was adjusted for adjusted for age, sex, ethnicity, educational attainment, income, population density, and IMD. Hazard ratio (HR), relative excess risk due to interaction (RERI), not a number (NA).

	Measures	Estimates	L95	U95	p-value
1	HR00	1.000	NA	NA	NA
2	HR01	1.245	1.142	1.358	0.000
3	HR10	1.036	0.982	1.092	0.195
4	HR11	1.279	1.180	1.388	0.000
7	Multiplicative scale	0.992	0.934	1.054	0.800
8	RERI	-0.001	-0.068	0.065	0.516

Table 20: Effect modification for pollution score. L95 and U95 correspond to the lower and upper limit of the 95% confidence interval. The model was adjusted for adjusted for age, sex, ethnicity, educational attainment, income, population density, and IMD. Additionally, here we excluded participants who lived less than 5 years (instead of 1 year) at baseline address. Hazard ratio (HR), relative excess risk due to interaction (RERI), not a number (NA).

11 Proportionality assumption check

We checked the proportionality assumption for Cox regression using Schoenfeld residuals. In figure 14 we plot the linear fit to the residuals obtained from the main model, using all-cause dementia as an outcome and with all pollutants, scaled by interquartile range, as exposure. Comparable results were obtained from single pollutant models, as exemplified in Figure 15 for $PM_{2.5}$. We found that proportionality assumptions were violated for age, sex, income, PM_{10}, $PM_{2.5}$, $PM_{a}bsorbance$ and NO_2 ($p < 0.05$), but that results were likely due to the high sample size: in fact, no clear violation was visible when plotting the residuals (Figure 16), especially for air pollutants. Moreover, the results did not change when regressing all-cause dementia on exposure to pollution score when stratifying the effect of age, sex, and income by time (Table 21): this indicates that our effect estimates for air pollution were likely constant over time-in-study, thus respecting the proportionality assumption. Here we split time in two using a cut-point at 12 years, based on the visual inspection of the order 3 polynomial fits, shown in Figure 17, as suggested by [12].

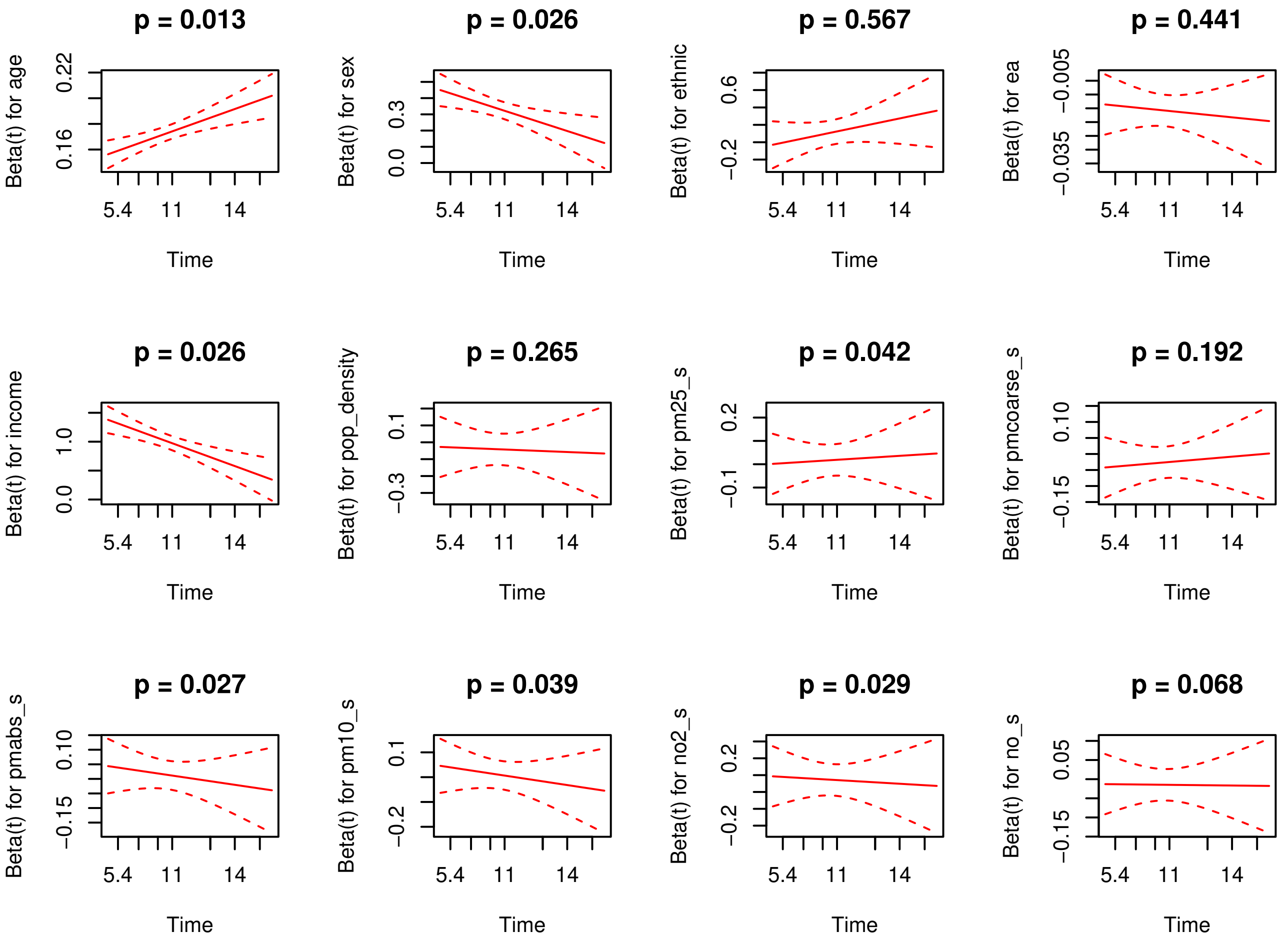

Figure 14: Linear fit, and associated p-values, to Schoenfeld residuals. The model was adjusted for age, sex, ethnicity, educational attainment, income, and population density, the outcome was all-cause dementia and the exposure included all air pollutants.

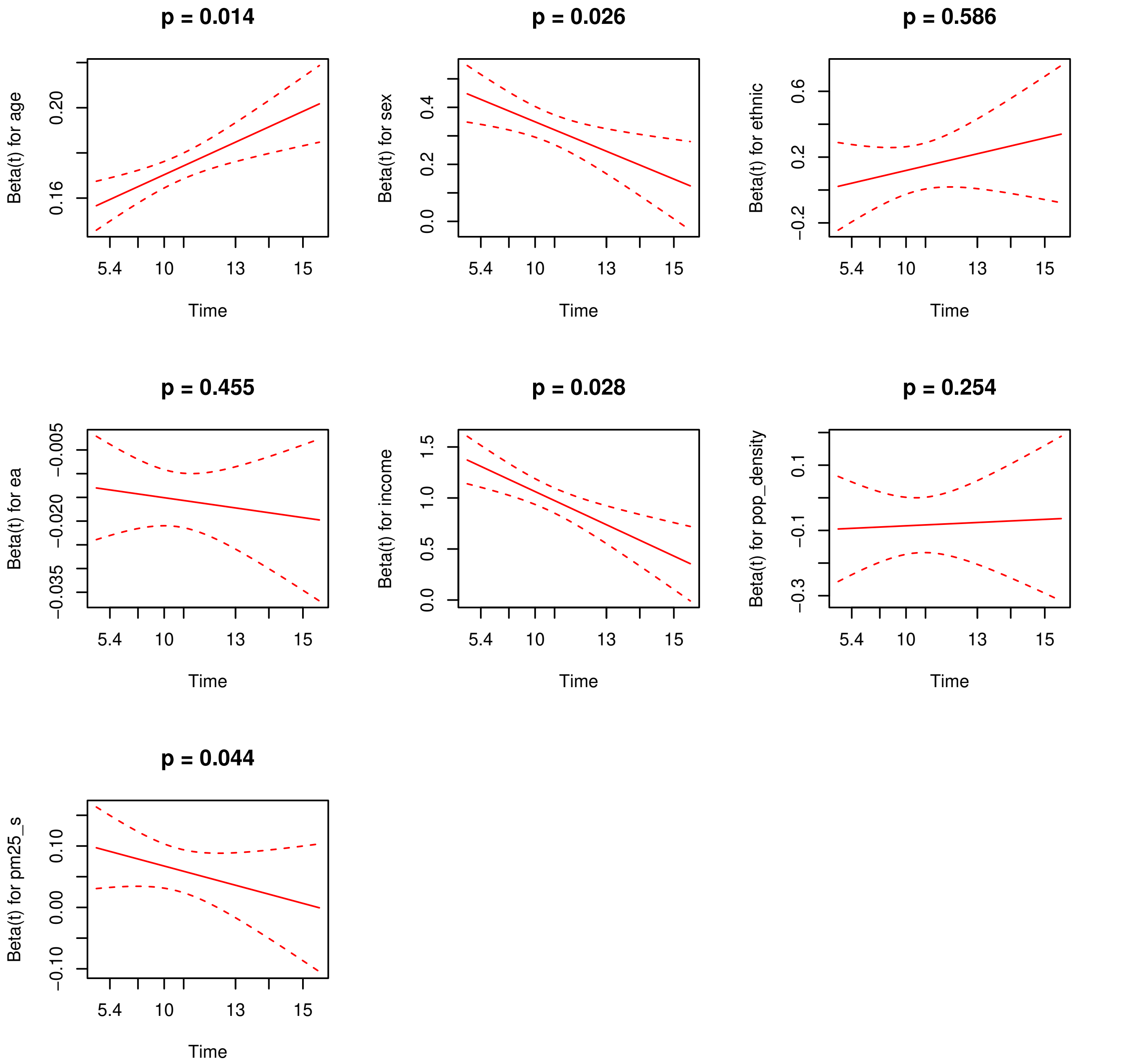

Figure 15: Linear fit, and associated p-values, to Schoenfeld residuals. The model is the same as in Figure14, but with only $PM_{2.5}$ as exposure.

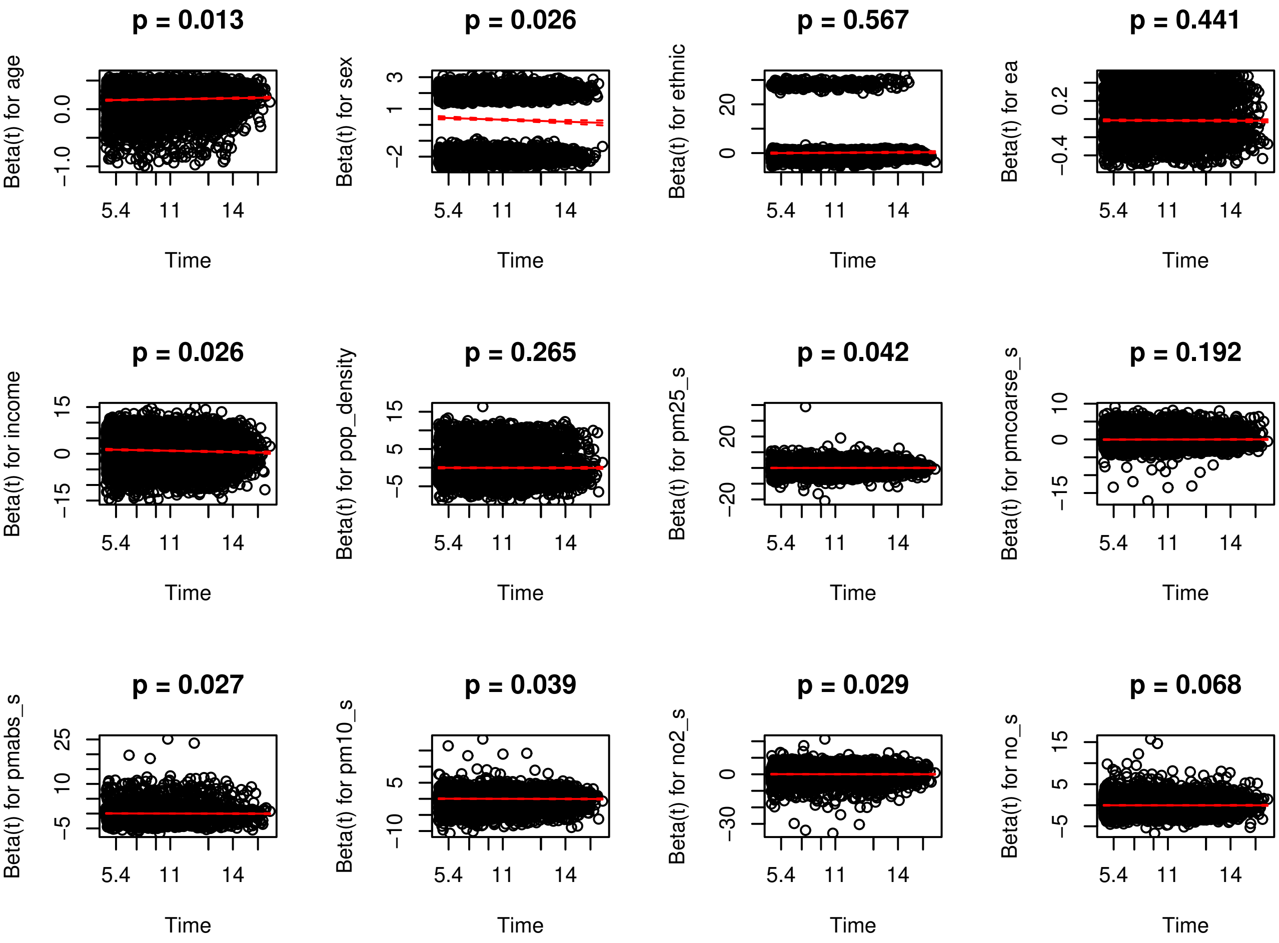

Figure 16: Plot of Schoenfeld residuals together with the linear fit. The model is the same as in Figure14

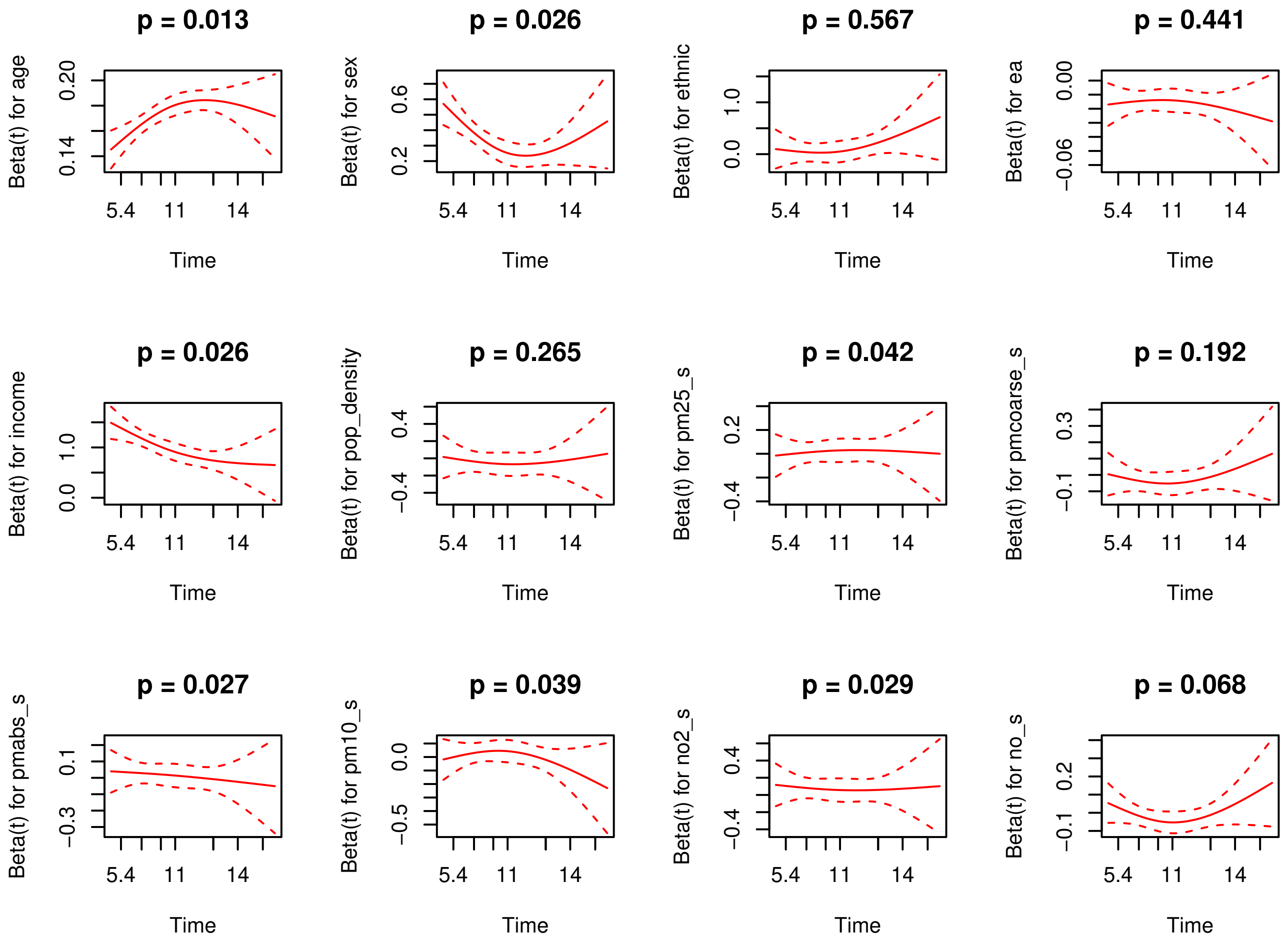

Figure 17: Degree 3 polynomial fit to Schoenfeld residuals. The model is the same as in Figure14. The fits highlight a consistent change in slope approximately around 12 years.

model	HR	L95	U95	p-value
pollution score	1.0348	1.0162	1.0537	<0.001
pollscore-age	1.0348	1.0162	1.0537	<0.001
pollution score-sex	1.0348	1.0162	1.0537	<0.001
pollution score-income	1.0349	1.0164	1.0538	<0.001

Table 21: Comparison of hazard ratio for air pollution score for different models. Here we used the main model (adjusted for age, sex, ethnicity, education, income, and population density) with pollution score as an exposure as reference. The other models included respectively an interaction between time period and age, sex, and income. The hazard ratio for pollution score for the different models is invariant, suggesting that the effect estimates in our analysis were constant over time-in-study.

12 Air pollutants characteristics

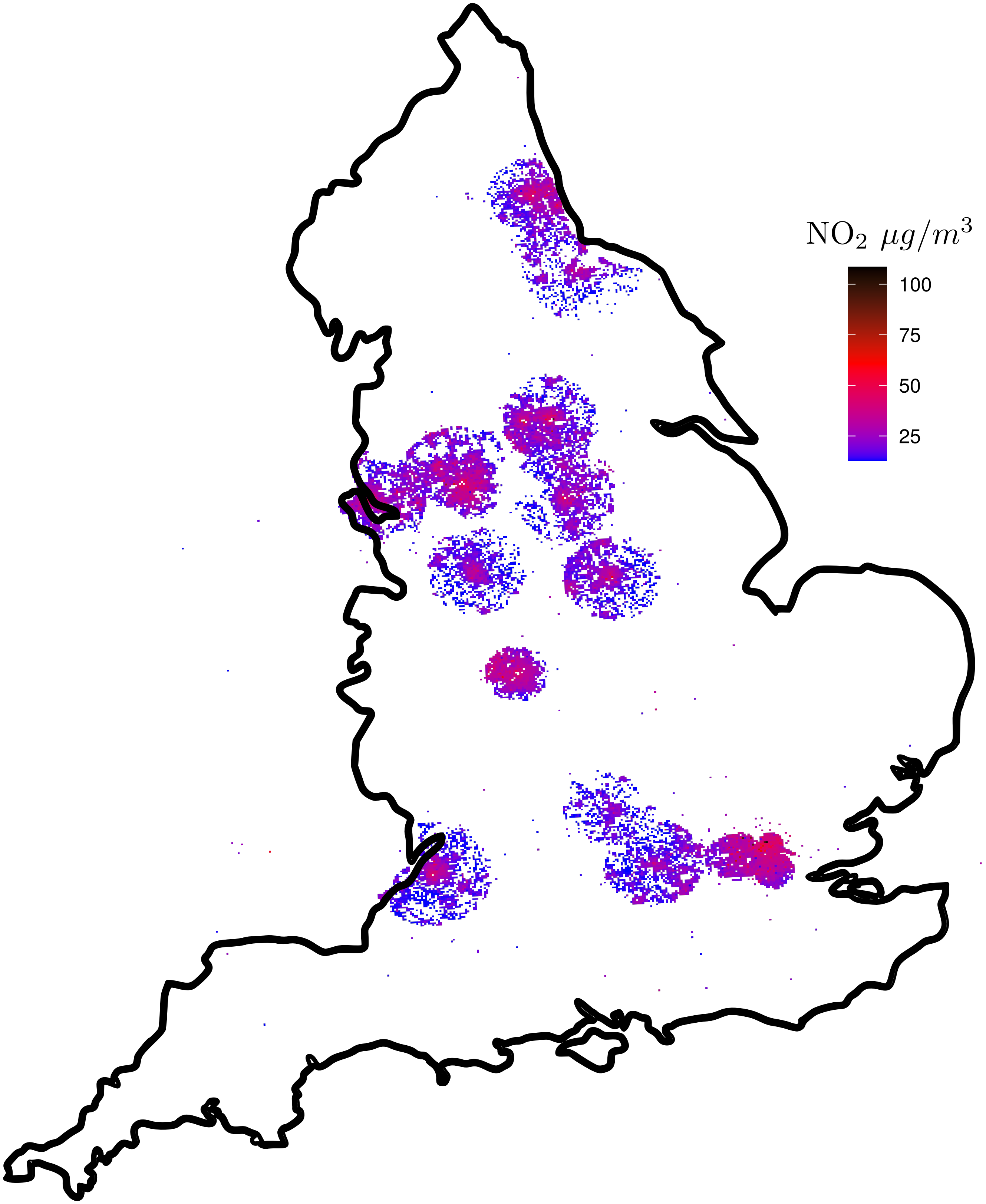

Figure 18: Spatial distribution of NO_2 exposure across participants considered in this study

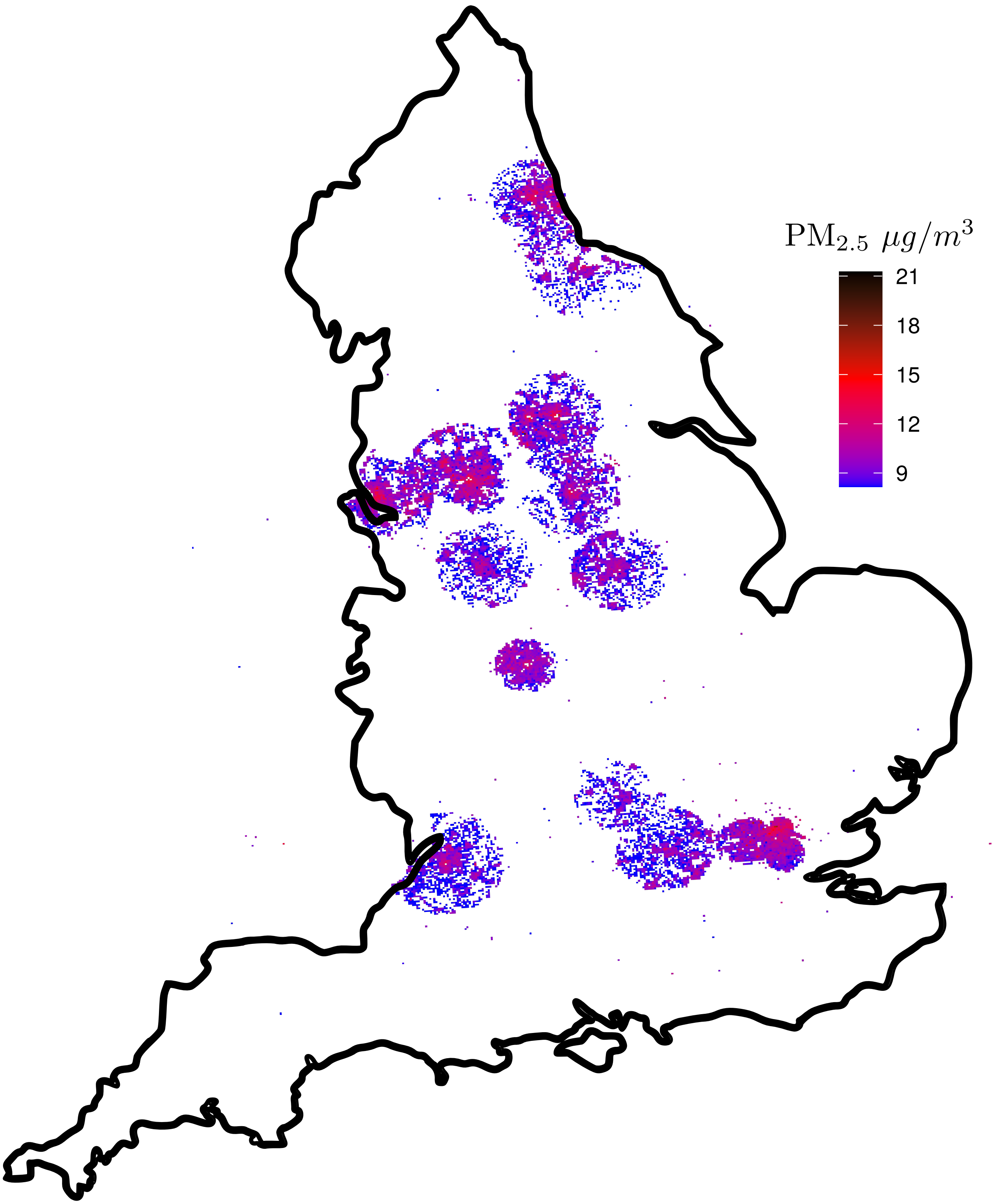

Figure 19: Spatial distribution of PM$_{2.5}$ exposure across participants considered in this study

Pollutant	Min.	1st Q	Median	Mean	3rd Q	Max.
$pm_{2.5}$	8.170	9.280	9.920	9.973	10.550	21.250
pm_{coarse}	5.570	5.840	6.100	6.411	6.620	12.820
pm_{abs}	0.830	.990	1.130	1.182	1.300	4.570
pm_{10}	11.78	15.23	16.02	16.21	16.98	30.65
NO_2	12.93	21.31	26.07	26.56	31.17	108.49
NO	0	11.61	15.94	17.23	20.53	160.06

Table 22: Distribution of air pollution data. Units are in $\mu g/m^3$. Q is quartile

	$pm_{2.5}$	pm_{coarse}	pm_{abs}	pm_{10}	no_2	no
$pm_{2.5}$	1.00	0.22	0.60	0.54	0.86	0.73
pm_{coarse}	0.22	1.00	0.42	0.81	0.20	0.24
pm_{abs}	0.60	0.42	1.00	0.56	0.75	0.49
pm_{10}	0.54	0.81	0.56	1.00	0.51	0.46
no_2	0.86	0.20	0.75	0.51	1.00	0.74
no	0.73	0.24	0.49	0.46	0.74	1.00

Table 23: Pearson correlation between air pollutants

	PC1	PC2	PC3	PC4	PC5	PC6
$pm_{2.5}$	-0.44	-0.30	0.14	-0.59	0.51	-0.29
pm_{coarse}	-0.29	0.71	0.12	0.14	0.50	0.36
pm_{abs}	-0.42	0.01	-0.77	0.34	0.07	-0.32
pm_{10}	-0.41	0.47	0.16	-0.30	-0.64	-0.29
no_2	-0.46	-0.33	-0.15	-0.13	-0.27	0.76
no	-0.41	-0.28	0.56	0.64	-0.04	-0.16

Table 24: Pollutant loads in the PCA. Only loads of the 1st eigenvector has similar magnitude as same sign.

	PC1	PC2	PC3	PC4
pm_{25}	-0.52	-0.21	0.65	-0.52
pmabs	-0.45	0.79	-0.31	-0.26
no_2	-0.54	0.05	0.22	0.81
no	-0.48	-0.57	-0.66	-0.11

Table 25: Pollutant loads in the restricted PCA. Only loads of the 1st eigenvector has similar magnitude as same sign.

13 Variation inflation factor

Independent variables	model 1	model 2	model 3
age	1.107	1.118	1.118
sex (male)	1.016	1.018	1.018
ethnicity (other)	1.046	1.048	1.047
educational attainment	1.219	1.244	1.249
income (18-31k)	1.264	1.294	1.309
income (31-52k)	1.337	1.379	1.396
income (52-100k)	1.271	1.305	1.321
income (>100k)	1.078	1.087	1.085
population density (rural)	1.496	1.497	1.509
IMD (2^{nd}q)	NA	1.584	1.588
IMD (3^{rd}q)	NA	1.674	1.677
IMD (4^{th}q)	NA	2.076	2.067
NO_2	7.757	7.909	7.895
NO	2.725	2.701	2.709
PM_{10}	5.384	5.521	5.551
PM_{25}	4.645	4.739	4.720
PM_{abs}	2.810	2.828	2.837
PM_{coarse}	4.396	4.457	4.481

Table 26: Variance inflation factor (VIF). The table displays the VIF associated with each independent variable in cox models with all-cause dementia as outcome and all pollutants included. Model 1 corresponds to the model used in the primary analysis; model was 2 additionally adjusted for by IMD; model 3 used only participants with $\geq$ 5 years at baseline residence. The VIF of the pollutants, in particular NO_2, indicates that their simultaneous inclusion in a model may cause moderate multicollinearity issues; on the other hand, there is little concern for including all covariates in the same model.

References

[1] Marloes Eeftens et al. "Development of land use regression models for PM2. 5, PM2. 5 absorbance, PM10 and PMcoarse in 20 European study areas; results of the ESCAPE project". In: *Environmental science & technology* 46.20 (2012), pp. 11195–11205.

[2] Rob Beelen et al. "Development of NO2 and NOx land use regression models for estimating air pollution exposure in 36 study areas in Europe–The ESCAPE project". In: *Atmospheric Environment* 72 (2013), pp. 10–23.

[3] Josef Cyrys et al. "Variation of NO2 and NOx concentrations between and within 36 European study areas: results from the ESCAPE study". In: *Atmospheric Environment* 62 (2012), pp. 374–390.

[4] Mengying Wang et al. "Joint exposure to various ambient air pollutants and incident heart failure: a prospective analysis in UK Biobank". In: *European heart journal* 42.16 (2021), pp. 1582–1591.

[5] Xiang Li et al. "Obesity and the relation between joint exposure to ambient air pollutants and incident type 2 diabetes: a cohort study in UK Biobank". In: *PLoS Medicine* 18.8 (2021), e1003767.

[6] Aysu Okbay et al. "Polygenic prediction of educational attainment within and between families from genome-wide association analyses in 3 million individuals". In: *Nature genetics* 54.4 (2022), pp. 437–449.

[7] Stylianos Kephalopoulos et al. "Advances in the development of common noise assessment methods in Europe: The CNOSSOS-EU framework for strategic environmental noise mapping". In: *Science of the Total Environment* 482 (2014), pp. 400–410.

[8] Rachael A Hughes et al. "Accounting for missing data in statistical analyses: multiple imputation is not always the answer". In: *International journal of epidemiology* 48.4 (2019), pp. 1294–1304.

[9] Alexander P Keil et al. "A quantile-based g-computation approach to addressing the effects of exposure mixtures". In: *Environmental health perspectives* 128.4 (2020), p. 047004.

[10] Hui Zou and Trevor Hastie. “Regularization and variable selection via the elastic net”. In: *Journal of the Royal Statistical Society Series B: Statistical Methodology* 67.2 (2005), pp. 301–320.

[11] Caroline Carrico et al. “Characterization of weighted quantile sum regression for highly correlated data in a risk analysis setting”. In: *Journal of agricultural, biological, and environmental statistics* 20.1 (2015), pp. 100–120.

[12] Zhongheng Zhang et al. “Time-varying covariates and coefficients in Cox regression models”. In: *Annals of translational medicine* 6.7 (2018).
